# Adoption of paediatric and neonatal pulse oximetry by 12 hospitals in Nigeria: a mixed-methods realist evaluation

**DOI:** 10.1136/bmjgh-2018-000812

**Published:** 2018-06-26

**Authors:** Hamish R Graham, Ayobami A Bakare, Amy Gray, Adejumoke Idowu Ayede, Shamim Qazi, Barbara McPake, Rasa Izadnegahdar, Trevor Duke, Adegoke G Falade

**Affiliations:** 1 Centre for International Child Health, University of Melbourne, MCRI, The Royal Children’s Hospital, Parkville, Victoria, Australia; 2 Department of Paediatrics, University College Hospital, Ibadan, Nigeria; 3 Department of Paediatrics, University of Ibadan, Ibadan, Nigeria; 4 Department of Maternal, Newborn, Child and Adolescent Health, World Health Organization, Geneva, Switzerland; 5 Nossal Institute for Global Health, University of Melbourne, Melbourne, Victoria, Australia; 6 Bill and Melinda Gates Foundation, Seattle, Washington, USA

**Keywords:** child health, pneumonia, intervention study, other diagnostic or tool, qualitative study

## Abstract

**Introduction:**

Pulse oximetry is a life-saving tool for identifying children with hypoxaemia and guiding oxygen therapy. This study aimed to evaluate the adoption of oximetry practices in 12 Nigerian hospitals and identify strategies to improve adoption.

**Methods:**

We conducted a mixed-methods realist evaluation to understand how oximetry was adopted in 12 Nigerian hospitals and why it varied in different contexts. We collected quantitative data on oximetry use (from case notes) and user knowledge (pretraining/post-training tests). We collected qualitative data via focus groups with project nurses (n=12) and interviews with hospital staff (n=11). We used the quantitative data to describe the uptake of oximetry practices. We used mixed methods to explain how hospitals adopted oximetry and why it varied between contexts.

**Results:**

Between January 2014 and April 2017, 38 525 children (38% aged ≤28 days) were admitted to participating hospitals (23 401 pretraining; 15 124 post-training). Prior to our intervention, 3.3% of children and 2.5% of neonates had oximetry documented on admission. In the 18 months of intervention period, all hospitals improved oximetry practices, typically achieving oximetry coverage on >50% of admitted children after 2–3 months and >90% after 6–12 months. However, oximetry adoption varied in different contexts. We identified key mechanisms that influenced oximetry adoption in particular contexts.

**Conclusion:**

Pulse oximetry is a simple, life-saving clinical practice, but introducing it into routine clinical practice is challenging. By exploring how oximetry was adopted in different contexts, we identified strategies to enhance institutional adoption of oximetry, which will be relevant for scale-up of oximetry in hospitals globally.

**Trial registration number:**

ACTRN12617000341325.

**Key questions**

**What is already known?**

- Pulse oximetry is a cost-effective clinical tool that could prevent more than 150 000 child pneumonia deaths annually if it were more available in hospitals globally.
- Currently, there are no data evaluating how to introduce oximetry to hospitals where it is not yet available.

**What are the new findings?**

- Our study demonstrated successful institutional adoption of oximetry, revealing new lessons about how oximetry practices are adopted and how to promote oximetry more effectively.

**What do the new findings imply?**

- Oximetry can be successfully introduced into routine paediatric and neonatal care, but it requires organisational and individual behaviour change.
- We propose practical strategies that clinicians, managers and policy makers in low-resource settings can use to accelerate the scale-up of oximetry.

## Introduction

Hypoxaemia is a common and life-threatening complication of pneumonia, malaria, sepsis and many other conditions, affecting approximately 10%–15% of children and 20% of neonates admitted to hospitals globally.^1^ Hypoxaemia is difficult to detect clinically, and reliance on the WHO signs of hypoxaemia misses at least 20%–40% of cases.^2–5^

Pulse oximetry is the standard non-invasive method of detecting hypoxaemia and is increasingly recognised as an essential tool for the evaluation and management of sick children.^6^ Evidence from a recent systematic review showed that oximetry can improve mortality, admission practices and length of stay of children with hypoxaemia.^7^ A recent modelling study estimated that oximetry (together with access to oxygen therapy) could prevent up to 148 000 deaths from childhood pneumonia in 15 high-mortality countries at relatively low cost (US$2.97–U$52.92 per disability-adjusted life year averted).^8^

Recent advances in oximetry technology and reduced cost have made oximetry more accessible for hospitals in low-resource settings.^9^ Feasibility studies have shown that nurses and community health workers are able to use pulse oximeters effectively.^10 11^ However actual use of oximetry remains low—even when pulse oximeters are available.^12–16^ Experience from previous oximetry implementation projects suggests that while oximetry itself is not complicated, integrating oximetry into routine care practices is complex,^17–19^ requiring substantial behaviour change, and being influenced by a range of individual, social and environmental influences.^20^

We introduced oximetry into the paediatric and newborn wards of 12 Nigerian hospitals, as part of the Nigeria Oxygen Implementation project, a stepped-wedge trial seeking to improve the provision of oxygen to children and newborns.^21^ We derived our overall programme theory from our realist review of 20 oxygen therapy projects from 15 countries.^13^ In brief, we postulated that our intervention would increase oximetry knowledge/skills, improve access to pulse oximeters, stimulate social/cultural change, deliver observable benefits to patients, and thereby enhance motivation for users to sustain new practice until it became a personal habit and social norm.^13^

We conducted this evaluation to better understand how our intervention influenced oximetry adoption, and to inform future efforts to scale up oximetry in Nigeria and globally. Specifically, we aimed to understand (1) whether our oximetry intervention worked (and to what extent), (2) how healthcare workers (and hospitals) adopt oximetry practices, and (3) why the pattern of adoption varies under different circumstances.

## Methods

The Nigeria Oxygen Implementation project evaluation includes quantitative impact evaluation, process and technical evaluations, and mixed-methods assessment of programme theories using realist evaluation.^21^ This paper focuses on a realist evaluation of oximetry adoption in the 12 participating hospitals.

We used realist evaluation to enable a nuanced exploration of how our intervention changed oximetry behaviour and why this may have varied between different hospital contexts. Realist evaluation is a theory-based approach to evaluation that seeks to understand the ‘mechanisms’ through which an intervention generates an outcome in a particular context—‘what works, how, why, for whom, to what extent and in what circumstances’.^22 23^ By definition, mechanisms are causal processes (ie, responses to the intervention and/or participants’ resources) that are only activated in particular contexts (thus described as the realist principle of generative causation).^22 24^ The realist approach is particularly useful in the evaluation of complex interventions (like a multifaceted oxygen project),^23 25^ which typically work through multiple mechanisms and are influenced by a range of contextual factors (including social systems and structures).

### Setting

We conducted this study in 12 secondary-level hospitals in seven urban areas of south-west Nigeria between 1 January 2014 and 30 April 2017 (which coincided with a national economic recession^26^). These hospitals included government and mission hospitals of varying sizes, selected to be representative of secondary health facilities that admitted children.^12 21^ Hospitals were typically fee-paying, staffed by doctors and nurses (variable paediatric expertise), well stocked with medications but had poor power supply and oxygen availability (table 1, additional details in online supplementary appendix 1).

10.1136/bmjgh-2018-000812.supp1 Supplementary data

**Table 1:** Baseline characteristics, admissions and pulse oximetry adoption patterns of 12 secondary-level hospitals in south-west Nigeria

	H1	H2	H3	H4	H5	H6	H7	H8	H9	H10	H11	H12
Hospital type	Mission	Mission	State	State	State	Mission	State	State	State	Mission	Mission	State
Paediatric beds	**70**	**32**	**25**	**36**	**60**	**20**	**48**	**46**	**13**	**63**	**14**	**36**
Child+neonatal	(40+30)	(20+12)	(21+4)	(16+20)	(44+16)	(15+5)	(20+28)	(22+24)	(9+4)	(38+25)	(12+2)	(26+10)
Hospital staffing												
Access to paediatrician	Yes*	No†	Yes	Yes	Yes	Yes	Yes	No	No†	Yes*	Yes*	No†
Doctors—entire hospital	4	4	2	11	17	5	16	12	7	6	6	7
Nurses—child/newborn wards (number of paediatric-trained nurses)	18	7	16 (2)	33 (3)	62	9 (2)	26	31	11	18	4	26
Hospital oxygen supply												
Oxygen cylinders	Yes‡	Yes‡	Yes‡	Yes	Yes	Yes‡	Yes§	Yes	Yes‡	Yes	Yes‡	Yes‡
Oxygen concentrators	Yes¶	No	Yes¶	Yes¶	No	Yes	Yes¶	No	No	Yes¶	Yes¶	No
Pulse oximeters	0	0	0	0	3¶	1	0	0	0	0	1¶	0
Admissions (January 2014–April 2017)	**6730**	**1225**	**2423**	**5561**	**2911**	**1052**	**7475**	**2594**	**500**	**5904**	**1133**	**1017**
Child	2951	1065	2219	2952	2375	769	4437	1740	488	3745	1038	1016
Neonate	3779	160	204	2609	536	283	3038	854	12	2169	95	1
Adoption milestones (months**)												
>50% admissions	3	3	3	2	3	0	3	3	3	3	2	3
>90% admissions	4	5	5	10	4	0	13	3	13	5	3	3
6 months sustained >90%	9	17	10	–	12	6	–	19	18	15	8	–
Adopter category††	Fast	Medium	Fast	Slow	Medium	NA‡‡	Slow	Medium	Medium	Medium	Fast	Slow

Note: Neonate ≤28 days, child 29 days–15 years.

*Part-time.

†Family medicine consultant.

‡Not available in paediatric areas.

§Piped system connected to a large oxygen cylinder.

¶Present but not fit for use.

**Months to achieve targets, counted from introduction of oximetry (November 2015).

††Categories based on ranking of hospitals based on speed of achieving and sustaining high oximetry use (>90% of admissions).

‡‡H6 already practising oximetry.

NA, not applicable.

### Intervention

We introduced oximetry to all 12 hospitals in October–November 2015, as part of a stepped-wedge field trial seeking to improve access to and use of oxygen for children and neonates.^21^ We provided all hospitals with handheld pulse oximeters (Lifebox Foundation^9^) for use in their paediatric and neonatal wards. We conducted basic oximetry training for nursing and medical staff based on WHO guidelines^27 28^ using a brief (~1 hour) task-based teaching module with instructional video.^29^ Over the following 18 months, all hospitals received quarterly supervisory visits from the central project team, were encouraged to develop local multidisciplinary quality improvement teams and had access to an on-site project nurse for practical support. In addition, hospitals were randomised to receive an improved oxygen delivery system sometime over the subsequent 16 months (clusters of three hospitals, every 4 months). The improved oxygen system involved installation of concentrator-based oxygen delivery systems, technician training, and additional training for medical and nursing staff on the clinical use of oxygen.^21^ To encourage local ownership, we trained a small group of nurses and doctors (recommended by senior staff) as ‘master trainers’, then supervised them to run multiple half-day training sessions for colleagues (and encouraged them to lead periodic retraining).

### Study design

The first stage of our evaluation involved the identification of programme theories to explore, and articulation of potential context (C), mechanism (M) and outcome (O) configurations. For this, we reviewed the theories (and CMO configurations) identified in our realist review,^13^ and revisited the theoretical frameworks that informed our intervention (Social Cognitive Theory,^30^ Theoretical Domains Framework,^20^ COM-B Behaviour Change Wheel^31^), innovation adoption^32 33^ and organisational change management^34^ (also using these substantive theories to frame our discussion). We began the process of theory identification and CMO articulation prior to the intervention, and revised it continuously throughout the subsequent stages. Our initial list of potential theories was broad and imprecise (online supplementary appendix 2). From this, we prioritised particular theories for testing based on their perceived explanatory importance and their likely implications for future scale-up (with priorities changing as different perspectives emerged from data collection).

10.1136/bmjgh-2018-000812.supp2 Supplementary data

The second stage of our evaluation involved obtaining quantitative data on (1) oximetry practices and (2) user knowledge. Trained project nurses extracted data from the case notes of all children (aged <15 years) admitted to the hospitals during the study period using a standardised data collection form. We collected data on demographics, clinical presentation, assessment and management (including detailed data on oximetry and oxygen use). For a limited period of time, admitting nurses completed a supplemental nurse oximetry form recording their experience performing oximetry on individual patients (including problems encountered, time taken). We administered knowledge tests to all participants at the training workshops (pretraining and post-training). Knowledge tests included 12 true/false questions about oximetry (scored out of 12) and 5 best-choice scenarios that required interpretation of oximetry results (scored out of 20) (online supplementary appendix 3). We collected data on oximetry practices continuously throughout the study period, using summary results to repeatedly test programme theories and guide future lines of enquiry.

10.1136/bmjgh-2018-000812.supp3 Supplementary data

The third stage of our evaluation involved obtaining qualitative data on the (1) attitudes, beliefs and behaviours of users in relation to oximetry over time, and (2) user perspectives on how oximetry was adopted as a routine care practice (or not) in their hospital, and why this might have varied between different hospitals. We collected these data from two sources. First, we conducted focus groups with the 12 project nurses who were employed by our project team and embedded within participating hospitals. Project nurses were responsible for on-site data collection and general project support, giving them a unique observational perspective on how oximetry was adopted. We intentionally brought project nurses from different hospitals together to encourage deeper reflection on how causal mechanisms operated in different contexts to generate change in oximetry practice. Second, we interviewed 11 individual nurses and doctors from hospitals that we had identified as outliers (ie, preliminary data suggested they were relatively fast or slow adopters of oximetry practices) to clarify and explore ideas from the focus groups and understand how causal mechanisms were operating within particular contexts. We allowed for additional recruitment of interviewees if necessary to further refine programme theories.

A trained interviewer (HRG) facilitated the focus groups (in a private, non-hospital location) and individual interviews (in private offices at the respective hospitals), with a local assistant (AAB) in English (all participants were fluent in spoken and written English). Participants were recruited by the project manager (AAB), provided with written project information and gave written consent. We used a pilot-tested interview guide (online supplementary appendix 4) asking participants to recount experiences of oximetry introduction and adoption (or non-adoption) in relation to context.^35^ Our semistructured approach enabled us to articulate elements of our programme theory (existing and emergent) as they were raised, and facilitate discussion about how these played out in different contexts. We adjusted our approach between and during focus groups and interviews as our focus shifted from theory identification to theory refinement, recognising when we reached data saturation on some topics and ensuring we fully explored ideas and perspectives from different participants. We made audio-recordings and field notes of all interviews and focus groups, and returned transcripts to participants for comment and correction. We collected qualitative data in December 2016 (during our intervention period).

10.1136/bmjgh-2018-000812.supp4 Supplementary data

### Analysis

We followed an embedded approach to data analysis.^36^ We used quantitative data on oximetry practices to describe the pattern of oximetry adoption in participating hospitals over time (including variation between hospitals), and qualitative data (and other quantitative data) to explore the reasons behind this pattern of adoption—how and why oximetry practices changed under different circumstances. We triangulated inferences from the qualitative data with the quantitative data and additional field notes.

We used EpiData (EpiData Association, Odense, Denmark) for data entry and Stata V.12 for data cleaning and analysis. We calculated summary measures relating to oximetry practices and knowledge. We used linear regression to analyse the determinants of oximetry knowledge scores. We used logistic regression to analyse the determinants of success versus failure of oximetry attempts. To prevent contamination we nominated the month that an intervention was commenced in each hospital as a washout period, excluding these observations from analysis.

We used NVivo V.11 Pro (QSR International, Doncaster, Australia) to code and analyse the qualitative data. We used both inductive and deductive coding techniques, enabling us to explore potential mechanisms and influences we had already identified and to identify new themes emerging from the data. Two investigators (HRG, AG) performed initial data coding, and we used an iterative approach to identify, check and clarify emerging codes and themes, structuring them around the realist CMO framework.

We used an iterative approach to analysis, repeatedly reviewing and refining our programme theories using emerging data. For example, we used preliminary data on oximetry use (stage 2) to refine theories from our initial review (stage 1), and clarify questions for focus groups (stage 3). We then used data from the first focus group to further ascertain and refine theories, and identify new lines of questioning for the second focus group and areas that needed particular enquiry in interviews. We then explored the qualitative data more systematically, repeatedly testing inferences using additional data on oximetry use (stage 2) and supplementary field notes.

In our initial analysis we explored CMO configurations in each hospital (and individual ward), before constructing more generalisable ‘middle-range theories’ (data-derived concepts that are both sufficiently abstract to hold generally true and sufficiently concrete to be verifiable^37^). These middle-range theories were reviewed by the authorship group, alongside summaries of the coded qualitative data and summaries of the quantitative data, and repeatedly questioned, retested with the data and revised to reach agreement.

### Reporting

We followed the recommended reporting guidelines for realist evaluation (RAMESES II)^38^ and qualitative (Consolidated Criteria for Reporting Qualitative Research)^39^ studies.

## Results

### Participant characteristics

During the study period, 38 525 children were admitted to the participating hospitals, including 13 740 (35.7%) neonates (aged <28 days) (table 2). We obtained completed nurse oximetry forms for 2806 admissions. Approximately 900 healthcare workers attended oximetry or full oxygen training, and we obtained 740 completed pretraining tests and 633 post-training tests (19% missing/incomplete) (table 3).

**Table 2:** Participant characteristics and pulse oximetry use among children (<15 years of age) and neonates (≤28 days of age) at 12 secondary-level hospitals in south-west Nigeria (January 2014–April 2017)

	Preintervention	Pulse oximetry	Full oxygen system
	January 2014–October 2015	November 2015–step	Step–April 2017
Participant characteristics			
Total admissions	23 401	8856	6268
Neonate (%)	8799 (37.6)	2681 (30.3)	2260 (36.1)
Infant (%)	4418 (18.9)	1887 (21.3)	1124 (17.9)
Young child (%)	7704 (32.9)	3068 (34.6)	1952 (31.1)
Older child (%)	2480 (10.6)	1220 (13.8)	932 (14.9)
Sex, female:male (% female)	10 313:13 246 (43.8)	3804:5039 (43.0)	2804:3459 (44.8)
Neonate	3842:4866 (44.1)	1162:1508 (43.5)	977:1280 (43.3)
Infant	1911:2495 (43.4)	812:1074 (43.1)	536:588 (47.7)
Young child	3319:4359 (43.2)	1288:1779 (42.0)	878:1070 (45.1)
Older child	1124:1355 (45.3)	541:678 (44.4)	413:519 (44.3)
Median length of stay, days (IQR)	4 (2–6)	4 (2–6)	4 (2–7)
Neonate	4 (2–8)	5 (3–7)	5 (3–9)
Infant	4 (2–6)	4 (2–5)	4 (2–7)
Young child	3 (2–5)	3 (2–5)	3 (2–6)
Older child	4 (2–6)	3 (2–5)	4 (2–7)
Diagnosis, neonate (%)			
Neonatal sepsis	3921 (45.8)	1547 (58.1)	1075 (48.0)
Birth asphyxia	3444 (40.3)	955 (35.9)	884 (39.5)
Jaundice	2002 (23.4)	723 (27.2)	459 (20.5)
RDS/aspiration	277 (3.2)	101 (3.8)	53 (2.4)
Small/preterm	1458 (20.1)	524 (22.3)	423 (21.0)
Preterm	1286 (21.6)	489 (23.5)	388 (22.1)
Small (<2000 g)	814 (13.8)	255 (13.2)	231 (14.2)
LBW (1500–2000 g)	530 (9.0)	161 (8.3)	136 (8.3)
VLBW (1000–1500 g)	234 (4.0)	72 (3.7)	70 (4.3)
ELBW (<1000 g)	50 (0.9)	22 (1.1)	25 (1.5)
Diagnosis, child (%)			
Malaria	5146 (41.3)	2242 (36.9)	1515 (37.8)
Sepsis	4062 (29.7)	2212 (36.0)	1153 (29.1)
Diarrhoea	1719 (13.8)	920 (15.1)	478 (11.9)
Pneumonia	1855 (14.9)	734 (12.1)	539 (13.5)
Malnutrition	210 (8.4)	129 (9.9)	92 (10.1)
HIV/AIDS	18 (0.1)	8 (0.1)	4 (0.1)
Pulse oximetry use			
SpO_2_ documented on admission (%)	704 (3.0)	5868 (66.3)	5946 (94.8)
Neonate (%)	218 (2.5)	1829 (68.2)	2122 (93.9)
Infant (%)	104 (2.4)	1172 (62.1)	1075 (95.6)
Young child (%)	273 (3.5)	1962 (64.0)	1853 (94.9)
Older child (%)	104 (4.2)	905 (74.2)	894 (95.9)

Notes: Neonate ≤28 days, infant 29 days−11 months, young child 1–4 years, older child 5–14 years. Neonatal diagnoses based on recorded admission diagnosis and recorded admission weight and gestational age. Child diagnoses based on case definition, except for sepsis and HIV/AIDS (recorded admission diagnosis).

Step=hospitals were randomised to receive the full intervention beginning at one of four stepped time points (March 2016, July 2016, November 2016, March 2017).

ELBW, extremely low birth weight (<1000 grams); LBW, low birth weight (1500 to 1999 grams); RDS, respiratory distress syndrome; SpO2, oxygen saturation; VLBW, very low birth weight (1000 to 1499 grams).

**Table 3:** Change in participant pulse oximetry knowledge after basic pulse oximetry training and full oxygen training

	Basic pulse oximetry training		Full oxygen training	
	Pre	Post	Pre	Post
Participant demographics				
Number of participants	249	182	491	551
Mean age (years)	35.7	36.2	35.1	36.1
Sex (female:male)	174:71	130:40	427:61	475:74
Role (nurse:doctor)	149:65	120:29*	433:40	476:57
Mean service (years)	7.3	7.9	8.1	7.7
Paediatric	2.5	2.5	2.5	2.5
Neonatal	2.9	3.1	2.9	3
Oximetry knowledge test scores				
Mean score† (95% CI)				
True/false	6.4 (6.0 to 7.2)	6.8 (6.3 to 7.2)	6.9 (6.6 to 7.1)	8.7 (8.5 to 8.8)
Scenario	6.3 (5.8 to 6.9)	6.7 (6.1 to 7.4)	6.1 (5.7 to 6.4)	9.4 (9.1 to 9.8)
Adjusted effect‡ (95% CI)				
True/false		+1.7 (1.2 to 2.1)		+2.8 (1.8 to 3.8)
Scenario		+3.3 (2.6 to 4.1)		+4.8 (3.1 to 6.5)

*P value <0.05, using t-test for continuous outcomes and χ^2^ for binary outcomes.

†Participants completed pretraining and post-training tests, which included 12 true/false questions about pulse oximetry and 5 best-choice responses to scenarios (scored out of 20) (see online supplementary appendix 3).

‡Adjusted for type of training, type of healthcare worker, duration at hospital and duration of paediatric/neonatal experience.

We conducted two focus groups with all 12 of the project nurses and interviewed 11 individual hospital staff (8 nurses, 3 doctors) purposively sampled from selected ‘outlier’ hospitals (table 4). We had no loss of participants following enrolment, and participant review of transcripts confirmed that they accurately reflected the conversations.

**Table 4:** Characteristics of focus group participants (n=12) and interview participants (n=11)

Participant category	Median age (range)	Sex, % female (female:male)	Seniority, % senior (senior:junior)	Median years at hospital (range)
Project nurse (n=12)	27 years (22–53)	83 (10:2)	Not applicable	Not applicable
Hospital nurse (n=8)	46 years (28–56)	100 (8:0)	50 (4:4)	9 years (4–28)
Hospital doctor (n=3)	44.5 years (37–48)	100 (3:0)	67 (2:1)	7 years (4–14)

NB: Project nurses were employed by our project and embedded in the participating hospitals. Hospital nurses and doctors were local staff who received no payment from our project.

### Key outcome: oximetry practice

**Figure 1:**
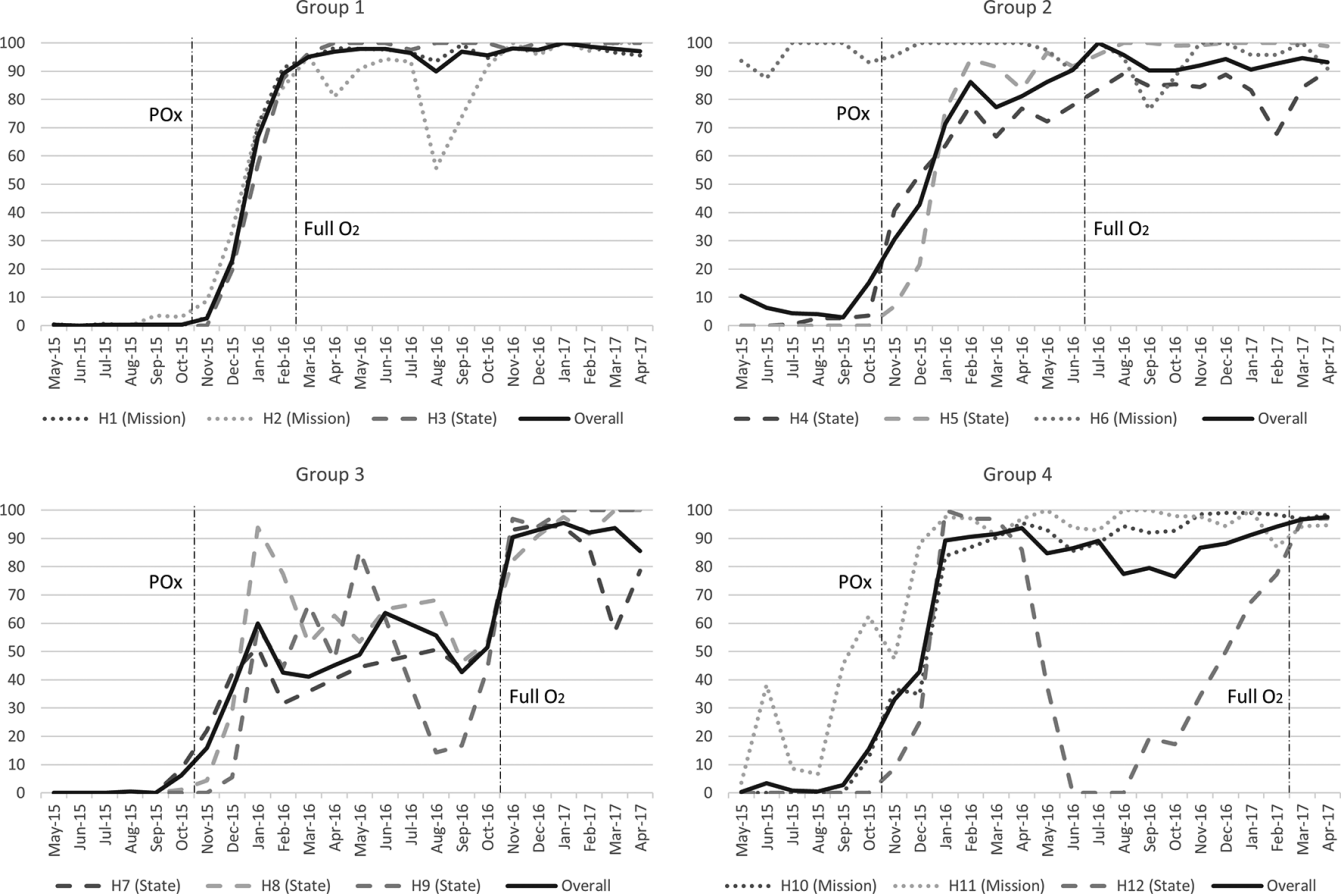
Pulse oximetry adoption: proportion of children and neonates with pulse oximetry documented on admission in 12 secondary-level hospitals in south-west Nigeria (May 2015–April 2017) showing the timing of introduction of pulse oximetry (POx) and full oxygen system (Full O_2_) for each group of hospitals. Five state hospitals (H4, H7, H8, H9, H12) were closed due to industrial action between 1 June and 10 August 2016.

Prior to our introduction of oximetry, 2.5% (218/8799) of neonates and 3.3% (481/14 602) of children had oximetry documented on admission—84% of readings were from a single mission hospital (H6) (table 2). Oximetry documentation increased to 68% (1829/2681) of neonates and 65% (4039/6175) of children following introduction of oximetry, and 94% (2122/2260) and 95% (3824/4008) following introduction of the full oxygen system. While all hospitals improved oximetry use within 2–3 months (reaching >50% of admissions), hospitals were more variable in reaching and sustaining high (>90%) levels of oximetry use (table 2, figure 1). For simplicity of reporting, we categorised hospitals as ‘fast’, ‘medium’ or ‘slow’ adopters based on their relative performance in achieving and sustaining high levels of oximetry use (table 1).

### Middle-range theories: how was oximetry adopted?

#### Enhanced knowledge and skill

**Figure 2:**
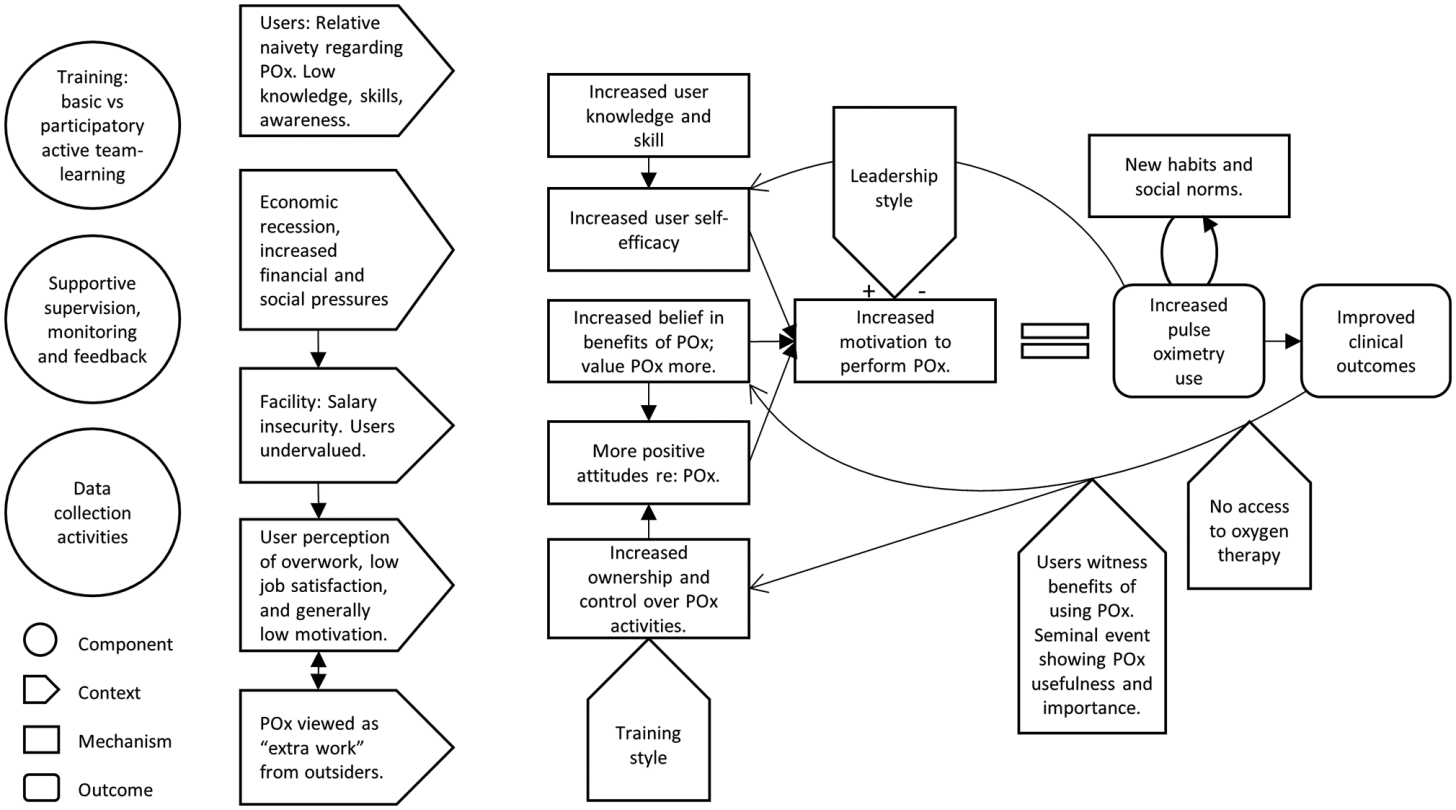
CMO configuration: knowledge, skills and attitudinal change. COM, context, outcome and mechanism; POx, pulse oximetry.

This theory posits that in the context of relative user naivety regarding oximetry (C), increased skills and self-efficacy (M) are important in increasing oximetry use (O), but are largely mediated by attitudinal change (see next theory) (figure 2).

Prior to our intervention, three hospitals (H5, H6, H11) had pulse oximeters in paediatric areas and two hospitals were doing oximetry semiregularly (H6, H11) (table 1). No hospitals had guidelines relating to the use of oximetry or oxygen. No hospitals included oximetry on nursing observation charts.

Interview participants reported that, while some doctors were familiar with oximetry, it was a fundamentally new practice for almost all nurses. They described being ignorant about the importance of oximetry and reported radical changes in people’s attitude and motivation as they acquired knowledge—particularly following the full oxygen training.

Will I call it negligence or will I call it, I don’t know. Maybe ignorance. Because we don’t know. We don’t know the importance of that pulse oximetry. Ah heh. I will say that many lives would have gone, you understand, because we don’t know. Assuming we know, we will have rescued so many lives. Now we know, we are able to put our, more efforts on to how to use it. (H7, Nurse 3 (N3))

Quantitative test scores support these qualitative observations. Baseline oximetry knowledge scores were low (table 3); multiple regression analysis revealed greater improvement following the full oxygen training.

Analysis of interhospital variation in oximetry test scores following oximetry training revealed a possible small association with oximetry practice: H2, H9 and H12 scored comparatively low and were average (H2, H12) or relatively slow (H9) adopters; H6 scored comparatively high and was the only hospital already routinely using oximetry. However, practice change was achieved successfully in all hospitals irrespective of test scores, suggesting that in a context of low oximetry experience, enhanced knowledge and skills are necessary, but not sufficient, to induce and sustain practice change.

#### Changing attitude: ‘I can’t do that’

This theory posits that in the context of low job satisfaction and motivation (C) and relative oximetry naivety (C), attitudes and beliefs about oximetry need to shift (M) before oximetry changes can be achieved and sustained (O) (figure 2).

Nurses responded to the introduction of oximetry to hospitals with mixed feelings. While some nurses were excited about the possibility of this new technology, most felt it was an additional burden to their already busy workload. Some nurses expressed negative feelings about the imposition of this new practice from perceived outsiders and felt they should be reimbursed for extra work (especially if their salaries were already overdue).

At [H7], when we actually brought the pulse oximeters, they were like, ‘we can’t do this, you know we have so many patients and we do the vital signs, check, and you want, ah what is this, we can’t do it’. In fact they had to pack it in their cupboard and lock it. (Project Nurse 9 (PN9))

This time of hardship, there is no salary, nothing, nothing. But you still give them extra work. I know to be do it, [but] they are not paying us. But if there were little bit of incentives [money]… (H12, N2)

They feel that they ought to be getting some money out of this project, but since they are not given anything. So then they thought that maybe the CC [Chief Consultant] or the Chief Matron in charge is collecting all the money, but they are not giving the junior staff this money, so they don’t need to do it. (PN11)

Despite widespread reservations about oximetry over the first few months, most hospitals showed substantial improvement in oximetry practices (figure 1). However, until hospitals achieved widespread attitudinal change, oximetry practices remained fragile and easily set back by events such as the relocation of a single key person (eg, H8, March 2016) or industrial action (eg, H7, H8, H9, H12, July 2016).

#### Changes with experience

Participants reported that early behaviour change required consistent external motivation (eg, reminders from senior staff and visits by project team) to overcome the strong negative attitudes and misconceptions. However, as nurses gained experience using oximetry, they also gained appreciation for the role of oximetry, confidence in using oximetry and motivation to do oximetry routinely.

People really appreciated the fact that training happened… But after the training, it wasn’t so easy getting them to do it. It was, it started a little bit challenging because you have to go there and have to beg them to do it… But with time, over time, people started having a changing of, a change of attitude towards it. Started appreciating it more. Started doing it better. Though it took some months, like three months, for them to be able to adapt to the change. (PN1)

Nurses came to view oximetry as a practice that ‘made their work easier’, giving them greater confidence in assessing and monitoring sick children. Oximetry became accepted as an essential part of routine clinical care (and described by nurses as ‘the fourth vital sign’, alongside temperature, heart rate and respiratory rate) that was done automatically.

It’s funny. Because when it was introduced, ah, I thought, ‘How are we going to do it, will it be possible to use this thing?’. Due to the workload here, I thought ‘ah, I think this thing is a wasting of time’. But…when it was implemented, in fact, it was wonderful. When we start using it, it made the work easier for us. (H4, N1)

Initially you have to think about [doing pulse oximetry]. Oh, have I done it, or not done it? But presently, …you don’t need to cerebrate to do that. It is at the level of the spinal cord. (laugh) You don’t even think about it. You just know, oh that has to be done. Someone doesn’t need to ask you ‘Have you done it?’. It’s already part of us. We just do it. (H1, Dr1)

Acceptance was a gradual process, but some nurses recalled specific incidents that accelerated change, reporting that the biggest motivation to continue oximetry was seeing it makes a difference to patients.

I have this scenario… A baby was born okay, there was no issues. Just an hour later, the mother noticed the neonate’s legs were purple, blue. The mother was like… ‘Please come and see my baby, come and see my baby’. [The nurse] was not paying attention until the O&G [obstetrician and gynaecologist] doctor came out and said, ‘Ah ah, what is wrong. What are you doing? I thought they brought a pulse oximeter in this ward. Where is it?’. Now they had to bring it majestically from their cupboard. ‘So you are not using this, now look’. And they check it. And the oxygen saturation was as low as fifty-something percent [emphatic]. And immediately they rushed the baby to the nursery, they started oxygen administration and other treatment given [and the baby survived]. And since then, I noticed they have been using it. (PN2)

Conversely, oximetry without access to oxygen could be demotivating. Indeed, while all participating hospitals had major oxygen access issues, two hospitals (H9, H12) had zero access to oxygen for children. These two hospitals struggled the most to adopt oximetry prior to improvement of their oxygen systems and showed rapid improvement afterwards. While these hospitals were not the only to show this pattern of improvement, they were unique in attributing it to oxygen access.

With experience, nurses came to value oximetry as a technical tool (accurate hypoxaemia measurement) and a clinical decision-making aid (guiding oxygen therapy). In addition, many nurses recognised oximetry’s broader value to nursing care (eg, monitoring and early detection of problems), communication and education (eg, to explain to family members and colleagues), and workload (eg, provide heart rate without palpation).

#### Workload burden

Participants identified excessive workload, inadequate staffing and lack of time as the major barriers to routinely performing oximetry on patients. However, quantitative data show little correlation between staffing, admission rates or relative workload (admissions per nurse). The fastest adopters included a large and busy mission hospital (H1), a well-staffed medium-sized government hospital (H3) and a minimally staffed small mission hospital (H11). The slowest adopters included two large and busy government hospitals (H4, H7), and a small but well-staffed government hospital (H12).

When oximetry was attempted, it was successful 98.7% of the time and usually took less than 2 min (table 5). However, failure was more likely in neonates (especially if preterm/small, adjusted OR (aOR) 5.88, 95% CI 0.72 to 48.28) and those who were ‘very agitated’ (child: aOR 23.7, 95% CI 9.19 to 61.11; neonate: aOR 18.5, 95% CI 1.53 to 223.7). Participants reported that technical difficulties (eg, the challenge of getting accurate readings on uncooperative children) were frustrating and demotivating, suggesting the need for both adequate patient load to practise and become proficient and adequate time to help troubleshoot and help nurses acquire this new skill.

**Table 5:** Pulse oximetry practice characteristics at 12 secondary-level hospitals in south-west Nigeria

	Neonate	Infant	Young child	Older child	Overall
Pulse oximetry (%)	n=906	n=598	n=916	n=386	n=2806
Succeeded	900 (99.3)	588 (98.3)	896 (97.8)	385 (99.7)	2769 (98.7)
Failed	6 (0.7)	10 (1.7)	20 (2.2)	1 (0.3)	37 (1.3)
Number of attempts if success					
1 (%)	437 (49.3)	324 (56.3)	607 (68.6)	295 (78.0)	1663 (61.0)
2 (%)	258 (29.1)	153 (26.6)	207 (23.4)	60 (15.9)	678 (24.9)
3 or more (%)	191 (21.6)	99 (17.2)	71 (8.0)	23 (6.1)	384 (14.1)
Number of attempts if fail					
1 (%)	0 (0)	1 (10.0)	1 (6.7)	0 (0)	2 (6.5)
2 (%)	0 (0)	0 (0)	0 (0)	0 (0)	0 (0)
3 or more (%)	5 (100)	9 (90.0)	14 (93.3)	1 (100)	29 (93.6)
Time to get reading (min)					
Successful, median (IQR)	2 (1–4)	2 (1–3)	2 (1–2)	2 (1–2)	2 (1–3)
Failure, median (IQR)	5 (5–10)	5 (4–10)	5 (3–10)	5 (–)	5 (4–10)
Signs/symptoms if success					
Cool peripheries (%)	178 (20.0)	28 (4.8)	42 (4.7)	20 (5.2)	268 (9.7)
Very active/non-cooperative	133 (14.9)	105 (18.0)	141 (15.8)	39 (10.2)	418 (15.2)
Very agitated or upset (%)	21 (2.4)	34 (5.8)	59 (6.6)	19 (5.0)	133 (4.8)
Shivering (%)	9 (1.0)	4 (0.7)	16 (1.8)	9 (2.4)	38 (1.4)
Oedema of hands/feet (%)	3 (0.3)	3 (0.5)	5 (0.6)	4 (1.0)	15 (0.6)
Painted nails (%)	2 (0.2)	2 (0.3)	4 (0.5)	1 (0.3)	9 (0.3)
Signs/symptoms if fail					
Cool peripheries (%)	1 (16.7)	1 (11.1)	1 (5.6)	1 (100)	4 (11.8)
Very active/non-cooperative	2 (33.3)	4 (40.0)	13 (68.4)	0 (0)	19 (52.6)
Very agitated or upset (%)	1 (16.7)	4 (40)	11 (57.9)	0 (0)	16 (44.4)
Shivering (%)	0 (0)	0 (0)	0 (0)	0 (0)	0 (0)
Oedema of hands/feet (%)	0 (0)	0 (0)	0 (0)	0 (0)	0 (0)
Painted nails (%)	0 (0)	0 (0)	0 (0)	0 (0)	0 (0)

Notes: Neonate ≤28 days, infant 29 days−11 months, young child 1–4 years, older child 5–14 years. Oedema=swelling of hands or feet due to excess fluid in subcutaneous tissues (often due to low body protein from malnutrition or kidney disease).

The perceived ‘burden’ of oximetry may relate more to an expression of general overwork and underappreciation—where oximetry was one extra task that nurses were being asked to do without recompense. This was more evident in government hospitals, where many people had not been paid their salary for more than 3 months and were feeling the impact of economic recession. Government hospitals tended to adopt oximetry more slowly than mission hospitals, and oximetry practices regressed in several of these hospitals following industrial action (and facility closure) in June–July 2016 (most noticeably in two smaller state hospitals: H9, H12).

[H10] Hospital is [a Mission hospital]… They pay them. They take care of their staff. But in government hospitals, owing somebody [salary] for 8 months, the psychological effect is there. They are no more loyal. No more sincere (eager) in doing their job. So they were forced to [do oximetry]. They were not ready to [do oximetry willingly]. (PN11)

#### ‘Teach me WHY’—training for behaviour change

**Figure 3:**
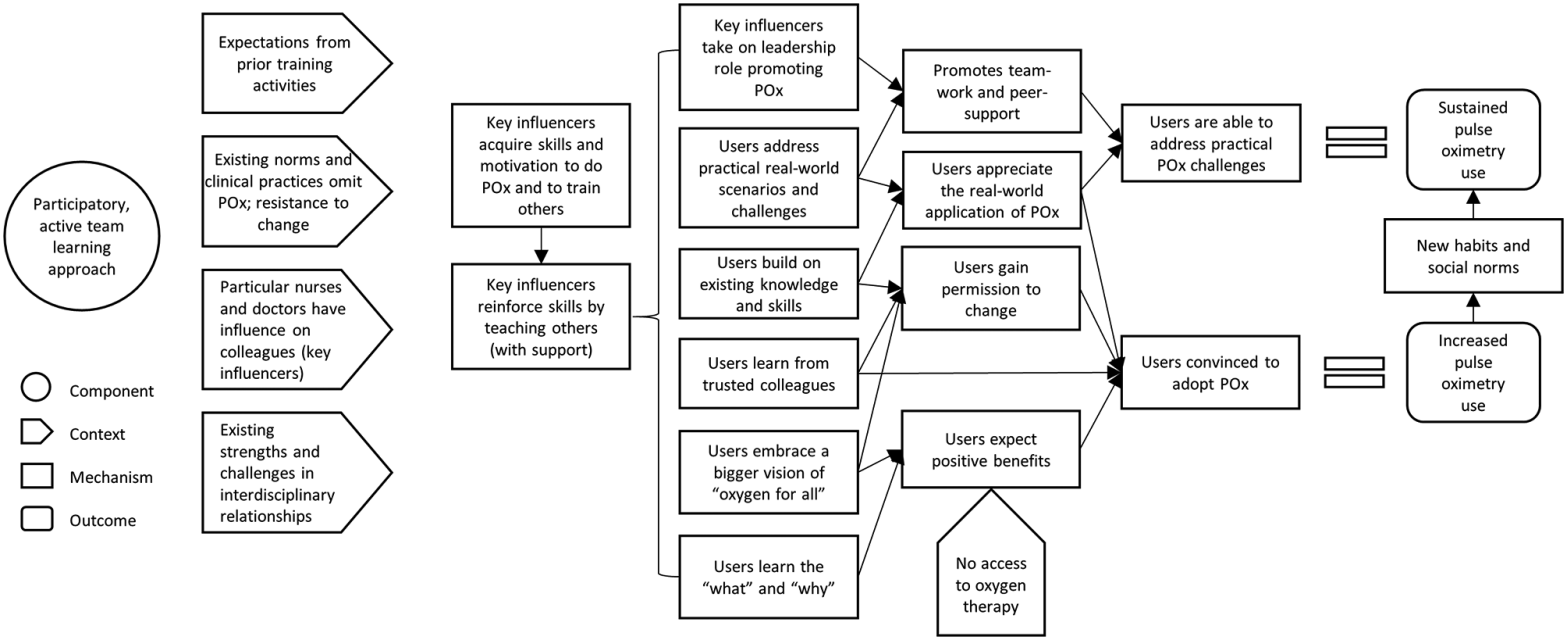
CMO configuration: training for behaviour change. CMO, context, mechanism, outcome; POx, pulse oximetry.

This theory posits that when particular influential members of the healthcare team (C) are supported to teach colleagues about oximetry using task-based participatory methods (I), other staff are more easily convinced (M) and supported (M) to adopt oximetry practices (O) (figure 3).

Participants reported that some training strategies were more effective in promoting behaviour change than others (box 1). First, the supervised training by local trainers enabled key influencers (particularly the head nurses) to acquire the knowledge, skill and motivation to use pulse oximeters effectively—then immediately demonstrate and reinforce these by training their junior colleagues while support was readily available.

In fact, one of the nurses that was trained in the ToT, the one that resisted us most, was the one that would train to retrain… She changed dramatically [after the full oxygen training] as if something removed the blindness from her and she saw light. She became our advocate. (PN11)

Second, participants described the need for “thorough sensitisation”, which addressed *why* oximetry was important and convinced users of the benefits (not simply taught them how to use a pulse oximeter or what they should be using it for). This reportedly enabled learners to embrace change and feel ownership. However, nurses from hospitals (H9, H12) that had zero access to oxygen for children reported that oximetry without oxygen was purposeless, and felt like they were “testing in vain”.

We need to tell them what they need to know, why they need to know it, and the outcome of them implementing it. So like really sensitising, because a lot of the time this thing has to do with our mind set. (PN1)

During the training, [the Project Manager] emphasised the vision, so that was what caught their attention. That it is the individual hospital that owns the project… So you see it as ‘my own’. (PN11)

We have no oxygen to give the patient. So they will be saying ‘why are we doing it? It is just for record purposes that we are doing it’. Even today we had one child, 81 percent. And we know they should go on oxygen [but] there is no oxygen. (H12, N2) (Prior to full oxygen system)

Third, the use of active learning techniques in a supportive team environment helped learners willingly embrace change to their beliefs and practice without humiliation or punishment.

The [full oxygen] training helped a lot. [The Project Manager] and his team they were fully on ground for days. You know, training, you know. With patients. It was…like a workshop, there was feedback, everybody was contributing. It is not a lecture that someone is delivering and saying ‘do it like this oh’. It was like a question and answer in a circle, and everybody was contributing, and people were happy, and people were seeing the logic. They saw pictures, they saw illustrated diagrams. We saw the machines live, how they will work… So it was exciting, we were eager to see the results that we would get from this project… We also had handbooks, and we had posters that we took to the ward for when we needed information we had charts that we would refer to, when we need more information as to what to do… Those things we pasted them on the wards, for quick reference. (H7, Dr1)

#### ‘Carry me along’—key influencers and leadership approach

**Figure 4:**
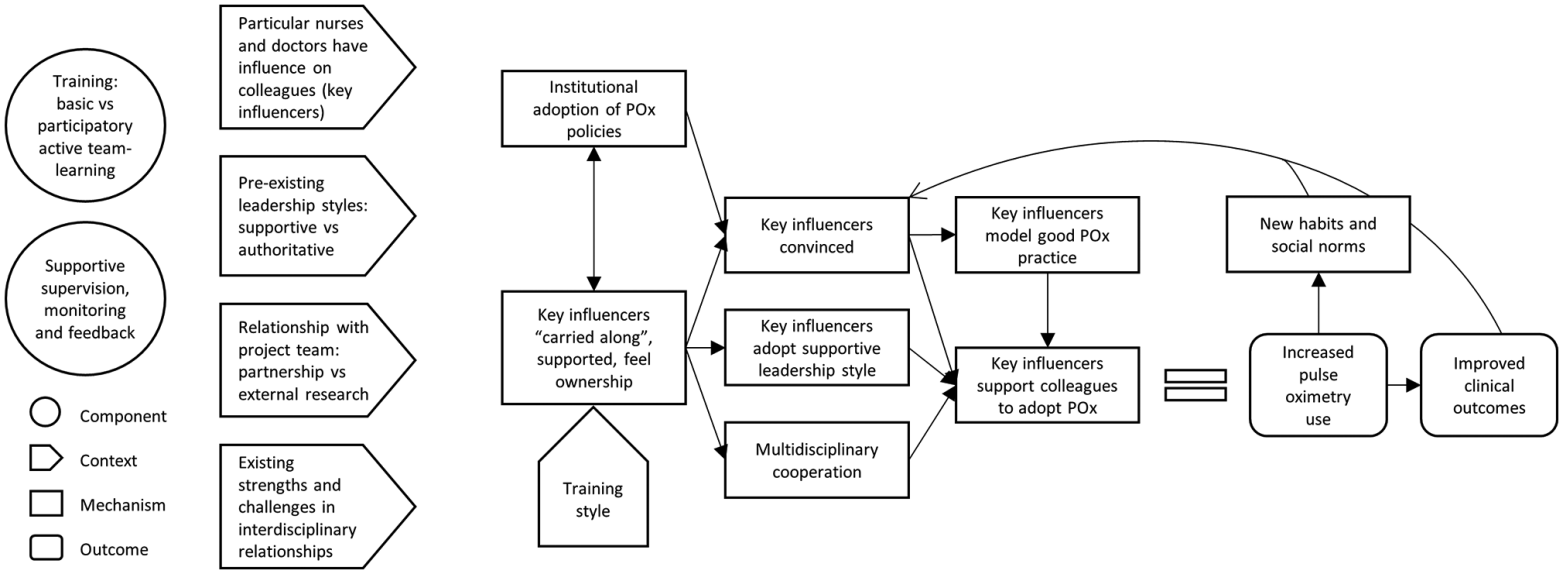
CMO configuration: leadership style and key influencers, ‘carry me along’. CMO, context, mechanism, outcome; POx, pulse oximetry.

This theory posits that when particular influential members of the healthcare team (C) are involved and given responsibility for oximetry implementation (I) in a supportive work environment (C), they provide practical support (M) and a modelled example (M) of oximetry use to colleagues, leading to enhanced adoption (O) (figure 4).

Participants reported the importance of ‘carrying along’ those with influence, particularly the head nurse on each ward. Where this was done well, these people became change makers, with the power, responsibility and motivation to enable their team to embrace oximetry as a valuable addition to their basic standard of care. Where the person of influence was not ‘carried along’, they frequently became the major obstacle to others using oximetry, and oximetry practices remained very poor.

But the major thing…[is to] recognise the core, important people that will drive this project. Because they are the ones that they are experienced, they have the position, they have the power, they have the authority. Because if they say that you cannot do it on their ward, then you can’t do it. So once you are able to carry them along, and they are interested, then the project is as good as done. (H4, Dr1)

Being ‘carried along’ meant more than just token involvement (although formal recognition and respect of authority was important). Being ‘carried along’ implied meaningful involvement in the implementation process, giving senior healthcare providers the skills and motivation to carry others along. This required project managers to impart responsibility and ownership to local leaders, while continuing to provide supervision and practical advice in a supportive and collegial way.

Participants emphasised that the style of engagement was paramount, requiring positive encouragement rather than a punitive approach.

You must be patient with everybody. You don’t get angry. You don’t get abusive, because they say ‘Ah, what is my own [duty]? Is it not to give drug to the patient and make the patient comfortable? I don’t have to do pulse oximetry’. Since they were not used to doing it before. But with patience and a lot of encouragement, and repeat of instructions, after a while everybody will be on board, and they will see the impact on patient management and things will be better. (H4, Dr1)

When senior doctors and nurses committed to working together, oximetry became a shared team activity that was valued, and would be done, by doctors and nurses alike. When senior nurses were not carried along, oximetry could become a source of tension between medical and nursing staff and result in nobody accepting responsibility for oximetry.

## Discussion

Pulse oximetry is a simple clinical practice using a simple technological device. However, adoption of oximetry by individuals and institutions is far more complex. We had the unique opportunity to evaluate the introduction of oximetry practices in a (almost) naïve environment. We observed relatively rapid adoption of oximetry practices, achieving coverage levels very close to 100% of admissions—substantially higher than has been reported in other projects.^17–19^ However, progress varied between hospitals and over time, and our realist evaluation revealed important lessons about how individual and institutional adoption of oximetry can be facilitated. We focus here on the implications for leaders and implementers at the hospital level, and seek to connect our key recommendations with existing behaviour change and innovation adoption frameworks.

First, many adoption-innovation frameworks emphasise the importance of leadership (both positively and negatively).^33^ In keeping with other studies, we found that a supportive and collegial leadership style enabled users to more easily modify their beliefs, attitudes and knowledge, and to sustain behaviour change (while punitive or authoritarian approaches resulted only in short-lived change).^40^ In addition, we found that opinion leaders were more likely to provide positive and supportive leadership if they were actively involved (‘carried along’) in implementation (ie, participatory approach). This is exemplified by the involvement of senior nurses and doctors as ‘master trainers’, whereby they gained modelling of supportive leadership approaches and took on real responsibility (and a prominent role) in the change process. Identifying and harnessing the support of the right influencers is difficult.^40^ The ‘master trainer’ approach facilitated this by involving multiple potential opinion leaders in a process that enabled the emergence of particular leaders.

Second, the introduction of oximetry requires more than the provision of equipment and training. Training is clearly useful, especially if it convinces learners ‘why’ they should change their practices and gives learners practical opportunities to use their newly acquired skills in their own work environment (box 1). Merrill’s^41^ ‘first principles’ provide a practical approach to this, promoting a task-based active learning approach structured around ‘activation’ (building on prior knowledge), ‘demonstration’ (showing), ‘application’ (practice and feedback) and ‘integration’ (application in their particular context). In addition to good training, healthcare workers also need reminders, encouragement from peers and seniors, and access to practical help as they master the new skill. Key influencers within the organisation are best placed to provide this supervision; however, external project officers may have a role in supporting and building capacity. We found Michie *et al*’s COM-B framework (and Behaviour Change Wheel^31^) useful to help leaders think more broadly about how they can stimulate changes in behaviour (B)—by increasing knowledge and skills (C, capability), and by creating a more enabling social and physical environment (O, opportunity) and giving people a compelling reason to act (M, motivation).

Box 1 **Strategies to enhance the effectiveness of pulse oximetry training (identified from focus groups and interviews)**

**Training will be more effective if it:**

- Communicates ‘why’ learners should change practice, not just ‘what’ they should do, and articulates this as a shared vision that learners can ‘own’.
- Connects new knowledge and practices with existing knowledge and practices, in an encouraging environment that fosters non-judgemental self-correction.
- Is hands-on and enhances problem-solving abilities so that learners can understand the real-world application and address particular challenges in the local context.
- Includes practical application for learners to apply newly acquired knowledge, including ongoing support to re-enforce learning and support the gradual process of change.
- Involves clinical leaders who will have ongoing influence through supervision, recognising their role, expertise and relationships with other staff.
- Builds teamwork through joint learning and problem-solving with colleagues.
- Uses job aids to aid learning and act as ongoing reminders in the clinical workspace.

Third, while oximetry has many attributes promoting its adoption (low *complexity*, high *observability*), we identified some strategies to boost its attractiveness to potential adoptees.^32^ Implementers can enhance the *relative advantage* of oximetry by promoting oximetry as a tool to ‘help healthcare workers do their jobs more easily’. Oximetry helps healthcare workers identify hypoxaemia and guide oxygen therapy, and helps them monitor sick patients, detect clinical deterioration, educate patients and junior staff, and promote positive physical interaction between patients and staff. Even the simple ability to simultaneously assess heart rate using oximetry was appreciated by many nurses. Communicating these broader benefits of oximetry may help overcome initial perceptions of ‘extra work’ (especially as many staff will only be convinced that oximetry is worth the effort after they have tried it and experienced the benefits themselves).

Implementers can enhance the *compatibility* of oximetry by embracing it as an essential vital sign for all admitted children. The WHO currently recommends that oximetry is performed on all patients presenting or admitted with respiratory illness, emergency signs or any sign of hypoxaemia.^27 28^ This is intended to target the highest risk patients and efficiently allocate scarce resources. However, previous studies that followed these recommendations struggled to attain usage levels above 50% even in the selected population of children with pneumonia.^17–19^ Our study suggests that the integration of oximetry practices into existing routines and professional identity was a major facilitator to adoption and likely contributed to sustainability. Moreover, we found that oximetry can be embraced as the ‘fourth vital sign’, appreciated as a practice that ‘makes our job easier’ and should not take users excessive time.

Finally, while oxygen and oximetry go hand in hand, most hospitals effectively adopted oximetry despite severe oxygen access challenges (even H9 and H12 made considerable progress despite zero oxygen access). Indeed, oximetry may have been particularly valued as it enables more rational use of this scarce resource (an idea we will explore further in our final evaluation).

We followed hospitals for 18 months after the introduction of oximetry and cannot report on long-term sustainability yet. However, oximetry has been effectively integrated into routine practice and no longer requires external support. Hospitals have successfully oriented and trained new staff. Hospital technicians have fixed minor equipment problems. Hospital managers have purchased additional pulse oximeters for use in other wards. In Nigeria’s user-pay system, hospitals identified financing to be the greatest threat to sustainability. To address this, hospitals have implemented small-scale oxygen insurance schemes whereby each patient pays a small ‘pulse oximetry’ fee which is used to maintain oximetry and oxygen equipment. Informal feedback suggests that this has been acceptable to patients and has generated adequate finance to cover all maintenance and repair costs (longer term evaluation is planned).

### Limitations

Using a mixed-methods realist approach to evaluation enabled a deeper and more nuanced exploration of how oximetry was adopted by focusing our attention on how change occurred and in what contexts. However, it is not possible to explore all possible mechanisms or contexts. We chose to focus on a theory level that would be most practically useful to hospital leaders seeking to introduce oximetry into their own environments. As such, we concentrate on the immediate hospital-level dynamics and do not delve deeply into many of the more specific individual, or broader socioeconomic and cultural factors.

The primary researcher (HRG) and interview assistant (AAB) had dual roles as project implementers and interviewers. Most of the staff participating in interviews and focus groups knew the interviewers and could have felt reluctant to criticise the project or their hospital. Postinterview debriefing suggested that respondents valued the opportunity to give feedback in a formal manner, were eager to discuss challenges and give suggestions for improvement, and familiarity with the interviewers helped facilitate discussion. Given the challenges of ‘insider’ researcher status,^42^ we sought to minimise bias in multiple ways. We framed interviews as an opportunity for us to hear from the ‘experts’, handing story-telling power to participants. The lead researcher made notes during and following interviews, recording his thoughts, questions and emotional reactions. We returned transcripts to participants for correction and feedback. We triangulated quantitative and qualitative data to verify findings. Multiple researchers contributed to coding, including one project ‘outsider’ (AG). The insider–outsider perspective also added value to our study, as interviewers had a wealth of informal feedback and observations that provided a practical understanding of discussions and provided a springboard for deeper exploration of ideas.

Our study benefited from a large sample size and minimal missing data. However, quantitative data on nurse’s oximetry experience involved a smaller population that may not have been fully representative of the larger study population. We obtained our qualitative data through focus groups and interviews with front-line users and implementers from 12 hospitals, intentionally selecting participants to capture a broad range of insights into how oximetry was adopted in different contexts. We faced some challenges when piloting our interview guide, as participants struggled to understand proposed programme theories. In response, we modified the interview guide to lead with more concrete questions, and used flexible interviewing techniques to facilitate discussion about the interplay between context, mechanism and outcomes.

Our study was conducted as part of a broader field trial and benefited from having dedicated project nurses and external supervision led by a dedicated project manager. However, this approach also had disadvantages as it created a perception that oximetry was being done for research purposes, rather than to help staff and patients, and raised expectations of financial incentives. This undoubtedly contributed to nurses’ perception they were doing extra work for others without personal gain and probably made local ownership harder to achieve.

Our findings are from paediatric areas within medium-sized Nigerian hospitals and may be less relevant outside this context. However, poor oximetry practices are common in hospitals throughout the world, particularly in low-income and middle-income countries,^12–16^ and our realist evaluation approach enables many lessons to be shared. Our study looked specifically at oximetry; however, our findings may be relevant to the introduction of other care practices, particularly those that involve the introduction of new health technologies.

## Conclusion

Pulse oximetry is a simple, life-saving clinical practice, but introducing it into routine clinical practice is challenging. By exploring how oximetry was adopted in different contexts, we identified strategies to enhance institutional adoption of oximetry, which will be relevant for the scale-up of oximetry in hospitals globally.
