# Correction: *Adoption of paediatric and neonatal pulse oximetry by 12 hospitals in Nigeria:a mixed-methods realist evaluation*

**DOI:** 10.1136/bmjgh-2018-000812corr1

**Published:** 2018-12-09

Graham HR, Bakare AA, Gray A, *et al*. Adoption of paediatric and neonatal pulse oximetry by 12 hospitals in Nigeria: a mixed-methods realist evaluation *BMJ Glob Health* 2018;3:e000812. doi: 10.1136/bmjgh-2018-000812

This article has been corrected since it first published. Owing to the image for Figure 3 and Figure 4 were back the front (ie, the titles are correct, but the wrong linking image). This is corrected in the latest version.

