# Supplementary material for: Adoption of paediatric and neonatal pulse oximetry by 12 hospitals in Nigeria: a mixed-methods realist evaluation
Source: BMJ Glob Health. 2018 Jun 26;3(3):e000812. doi: 10.1136/bmjgh-2018-000812 (PMC6035503; doi:10.1136/bmjgh-2018-000812)
Supplement: Supplementary data [file bmjgh-2018-000812supp001.pdf]

Hospital Descriptions

Contents

Hospital 1 (H1) 2
Hospital 2 (H2) 4
Hospital 3 (H3) 6
Hospital 4 (H4) 8
Hospital 5 (H5) 10
Hospital 6 (H6) 12
Hospital 7 (H7) 14
Hospital 8 (H8) 17
Hospital 9 (H9) 19
Hospital 10 (H10) 21
Hospital 11 (H11) 23
Hospital 12 (H12) 25

Hospital 1 (H1)

H1 is a long-established mission hospital in a city of approximately 1 million population. It is a busy hospital, with a particularly busy maternity and neonatal service. It is the only study hospital that has more neonatal admissions than maternity admissions. It provides a full range of medical, surgical, obstetric, paediatric and neonatal inpatient and outpatient services.

H1 is run by a non-medical Administrator, and has not recorded any periods of facility closure or industrial action. During times of industrial action in nearby government hospitals, the number of paediatric admissions to H1 has been noted to increase substantially.

H1 is a sprawling compound of mostly single story buildings. It has a very busy Neonatal unit beside the antenatal ward, with overflow cots in the entrance foyer. It has one large main Children's ward, as well as dedicated wards for LRTI, diarrhoeal disease, measles, and isolation. There is a new Emergency department. The OPD area is in a separate building.

Patient flow

Children presenting to the hospital will first be taken to the Children's OPD where they will be assessed by a Nurse and a Doctor and management will be initiated. If a child needs admission, they will be transferred to the Children's ward (or general Neonatal ward if <28 days old). The Children's OPD closes at 4pm. After this time children present to the Emergency Department (ED).

H1 receives referrals from government general hospitals, private hospitals, other mission hospitals, primary health centres. Children requiring referral from H1 are sent to tertiary hospitals (government and private). Transport is not available as the ambulance is too expensive for patients, so private transport is typically arranged.

H1 has 40 beds in the Children's ward, and 30 beds for Newborns (but are often above capacity).

Staffing

In the whole hospital there are 24 permanent doctors, 2 house officers, 148 nurses, 5 community health extension workers, and 54 health assistants. There are additional part-time Consultants who visit regularly.

There are 4 permanent doctors working in Paediatrics, all who are Family Medicine trainees. There are 2 visiting Consultant Paediatricians. Doctors work on call, with 4-5 doctors covering the whole hospital after hours.

There are 18 nurses who work with children and newborns, including 8 in Neonatal and 10 on the Children's wards. One nurse has Paediatric training; all nurses have double Nurse-Midwifery training. Nurses work three shifts to provide 24/7 cover (2 per shift minimum, 1 may be a Nurse assistant).

Oxygen and Pulse oximetry

Prior to our study intervention, H1 had access to oxygen concentrators and oxygen cylinders. However, there were no oxygen cylinders in the paediatric areas and the oxygen concentrators were not producing medical grade oxygen. There was sufficient oxygen tubing present, and nasal prong cannulas (mostly single use, only reused occasionally). Pulse oximetry was not in use in any paediatric areas, but they were routinely used in theatre (and broken oximeters were in the

emergency ward). Oxygen therapy was already being prescribed to many patients based on clinical signs. Oxygen was moderately expensive, costing N1000 (USD$5) per day.

H1 had several key influencers take on leadership roles from an early time. The Administrator and Chief Medical Directors were actively involved in nominating responsibilities for the oxygen project, and two senior registrars took on substantial responsibility from the beginning. The nurse unit managers in both the paediatric and neonatal areas were actively involved from very early on, however the Matron (head hospital nurse) was not involved until later. H1 developed an effective multi-disciplinary oxygen team that met regularly and had the authority to effect practical change.

Systems

The mains power supply (NEPA) is very poor, and it is common to go for weeks without receiving any power. The hospital relies mostly on generators. There are 6 diesel generators which can provide the entire hospital with power. However, due to diesel supply and expense they are used sparingly to supply certain areas selectively.

There is a Maintenance team on site, who work mostly with the generators and buildings. Repairs for medical equipment is done by one of the technicians who is training another younger technician. There is no preventive maintenance for the concentrators (or any other medical equipment).

H1 had reasonable clinical records and administrative records systems (including a computer record system).

Pharmaceutical supplies are well stocked, using a user-pay system (including essential antibiotics, vaccines, IV fluids, and injection equipment). Essential supplies were available (including bedside glucometer, thermometer, neonatal and adult scales). Infection control equipment was available on every ward (including wash basin, soap, sharps and waste disposal, sterile gloves, chlorhex/iodine, alcohol swabs). Cord clamps and Gentian Violet were available in Birth Suite, and a working Resuscitaire and resuscitation equipment but no neonatal mask.

X-ray facilities are available. Laboratory facilities are available (including Hb, blood group and cross match, urinalysis, CSF microscopy, smear microscopy for acid-fast bacilli/TB).

Education and quality of care

H1 has regular educational sessions, including residency training program sessions for Family Medicine trainees 2-4 times per week. There are weekly drug presentations by pharmaceutical companies. Nurses used to have a program, but not regularly now. H1 has a nursing training program on-site, and nursing students are present in the paediatric areas most days, and assist nurses perform basic clinical duties. Mortality and Morbidity (M&M) meetings used to be held regularly but were discontinued a long time ago.

No standard treatment guidelines are used in paediatrics.

Financial

Patients were charged nightly admission feeds, paid for all medications and consumables (including oxygen). Inability to afford care does not prohibit treatment, however discharge release may be delayed until payment is made. The hospital prides itself on affordability and does not charge a mandatory deposit. The approximate total cost to the family of a young child admitted for 3 days with pneumonia is N13,500 (USD$72.5).

Hospital 2 (H2)

H2 is a small mission hospital in a regional area with population less than 500,000. It is one of three hospitals in the area – the others being a government state hospital, and the Muslim hospital. H2 is a moderately busy hospital, providing most of the maternity and paediatric services in the area. It provides medical, basic surgical, obstetric, paediatric and neonatal inpatient and outpatient services.

H2 is run by an experienced family medicine consultant (Chief Medical Director), and has not recorded any periods of facility closure or industrial action. During times of industrial action in nearby government hospitals, the number of paediatric admissions to H2 has been noted to increase substantially.

H2 is a single-story complex, spread over multiple wings. It has a refurbished Children's ward, adjoining the Female medical ward. It has a separate wing that is still being renovated for the Male medical ward. A separate wing has Maternity and birth suites. Within the Stage 1 ward there is a small area for Neonates for the care of all babies <28 days.

Patient flow

Children presenting to the hospital will first be taken to the main OPD/triage/registration area at the front of the hospital. Here they are seen (together with adults) then transferred to the Children's ward if they need admission. This is the same during and after hours.

It receives referrals from private hospitals, PHCs, General Hospitals, and the State Hospital Saki. Children requiring referral are sent to tertiary hospitals in other cities. Transport is by private transport, there is no Ambulance available.

H2 has 20 beds in the Children's ward, and 12 cots for Neonate (in a small room adjacent to Maternity Stage 1 ward). The Maternity staff look after the neonates.

Staffing

For the whole hospital there are 11 permanent doctors, 3 house officers, 36 nurses, 1 community health officer, and 1 health assistant.

For the Children's and Women's ward there are 3 permanent doctors (including 1 Consultant Family Physician), and 1 house officer. Doctors work on call, with multiple doctors available after hours.

There are 7 nurses on the Children's/Women's ward, plus additional in Midwifery who work with newborns. No nurses have Paediatric training; most nurses have double Nurse-Midwifery training. Nurses work three shifts to provide 24/7 cover for the General wing.

Oxygen and pulse oximetry

Prior to our study intervention, H2 had access to oxygen concentrators and oxygen cylinders. However, there were no oxygen cylinders or concentrators in the paediatric areas and the oxygen concentrators in other parts of the hospital were not producing medical grade oxygen. Pulse oximetry was not in use in any paediatric areas. Oxygen therapy was occasionally prescribed to patients based on clinical signs. Oxygen was prohibitively expensive, N500 per hour (USD$60 per day). There was adequate oxygen tubing present, and nasal catheters were the main route of administration.

H2 had several key influencers take on leadership roles from an early time. The Chief Medical Director and the Director of Nursing Services were involved from the beginning, and provided

routine supervision. The ward matrons, at the instance of the Director of Nursing Services, also supervised pulse oximetry services for inpatients admissions.

Systems

The mains power supply (NEPA) is very poor, and it provides at best a few hours each day. The hospital relies mostly on generators. The large diesel generators are used for periods of 6 hours in the day and 3 hours in the evening. Small generators are used for specific purposes during the day (e.g. theatre).

There are technicians on-site who perform some basic medical equipment repairs.

H2 had reasonable clinical records and administrative records systems. H2 has regular educational sessions. Clinical meetings, Doctor' conference, monthly Ward conference, Maternity meetings. There are monthly Mortality and Morbidity (M&M) meetings for the whole hospital.

No standard treatment guidelines are used in paediatrics.

Pharmaceutical supplies are well stocked, using a user-pay system (including essential antibiotics, vaccines, IV fluids, and injection equipment). Essential supplies were available (including bedside glucometer, thermometer, neonatal and adult scales). Infection control equipment was available on every ward (including wash basin, soap, sharps and waste disposal, sterile gloves, chlorhex/iodine, alcohol swabs). Cord clamps and Gentian Violet were used, but there was no newborn resuscitation equipment available.

X-ray facilities are available. Laboratory facilities are available (including Hb, blood group and cross match, urinalysis, smear microscopy for acid-fast bacilli/TB, CSF microscopy).

Financial

Patients pay a daily admission fee, and pay for all medications and consumables (private on-site pharmacy), including oxygen. The approximate total cost to the family of a young child admitted for 3 days with pneumonia is N10,800 (USD$50.4).

Hospital 3 (H3)

H3 is a government state hospital in a city of approximately 1 million population. It provides a full range of medical, basic surgical, obstetric, paediatric and neonatal inpatient and outpatient services.

H3 is run by a specialist physician (Chief Medical Director). H3 has been badly affected by industrial action, being closed for approximately 20% of the time in the 18 months prior to the project period. During times of industrial action, H3 closes entirely and typically takes some weeks to resume full operational capacity after re-opening.

H3 is a single-story sprawling complex. It has one large Children's ward, and an adjacent smaller ward for Neonates (with overflow into the main Chidren's ward). A separate building houses the Emergency Department, offices, adult wards, and outpatient pharmacy.

Patient flow

Children presenting to the hospital will first be taken to the Children's OPD where they will be seen by a Doctor and treatment will be started. Those requiring admission will be sent to the ward. After 4pm the OPDs are closed, and children are seen via the Emergency Department (ED).

It receives referrals from private hospitals, PHCs, General Hospitals. Children requiring referral are sent to tertiary hospitals in neighbouring cities. Transport is available in a hospital ambulance, at a cost to patients – most will arrange private transport.

H3 has 21 beds in the Children's ward, and 4 cots for Newborns (in a small room adjacent to the main Children's ward, with overflow into the Children's ward).

Staffing

In the whole hospital there are 25 permanent doctors, (usually 12 house officers, but none at time of visit), 149 nurses, 2 public health nurses, 1 midwife (sole trained), and 3 health assistants.

There are 2 permanent doctors for Paediatrics, and 2 visiting Consultant Paediatricians. Doctors work on call, with 2 doctors covering the entire hospital after hours.

There are 16 nurses who work with children. Two nurses have Paediatric training; most nurses have double Nurse-Midwifery training. Nurses work three shifts to provide 24/7 cover (2 per shift).

Oxygen and pulse oximetry

Prior to our study intervention, H3 had access to oxygen concentrators and oxygen cylinders. However, there were no oxygen cylinders in the paediatric areas and the oxygen concentrators were not producing medical grade oxygen. Pulse oximetry wan not used anywhere in the hospital. Oxygen therapy was occasionally prescribed to patients based on clinical signs. Oxygen was moderately expensive, N1500 per day (USD$7.50). There was sufficient oxygen tubing present, and nasal prong cannula were available, and are washed and re-used.

H3 had slow initial uptake by key influencers. The Chief Medical Director initially delegated responsibility to a paediatrician. The changing moment was about 3 months post pulse oximetry introduction, when we involved the Apex nurse and the Chief Nursing Officer at the emergency ward while conducting supportive supervision. The Chief Nursing Officer took up the responsibility to perform pulse oximetry, and this served as role model for other nursing staff.

Systems

H3 has relatively poor infrastructure, mostly dependent on large diesel generators providing power to essential services during selected hours, and use of small petrol generators for emergencies at other times. H3 has highly trained onsite maintenance technicians/engineers, but limited maintenance procedures.

H3 had reasonable clinical records but poor central records systems, including collation of summary statistics into the State reporting books.

H3 has regular educational sessions. Weekly Grand Rounds, with different departments presenting topics. There is no dedicated paediatric teaching. There is weekly nursing teaching. Mortality and Morbidity (M&M) meetings are not held.

No standard treatment guidelines are used in paediatrics.

Pharmaceutical supplies are well stocked, using a user-pay system (including essential antibiotics, vaccines, IV fluids, and injection equipment). Essential supplies were available (including bedside glucometer, thermometer, neonatal and adult scales).

Infection control equipment was available on every ward (including wash basin, soap, sharps and waste disposal, sterile gloves, chlorhex/iodine, alcohol swabs). Cord clamps and Gentian Violet were not used (use cord in alcohol prepared by nurse), and a working Resuscitaire with neonatal resuscitation equipment (but no neonatal mask).

X-ray facilities are available. Laboratory facilities are available (including Hb, blood group and cross match, urinalysis, smear microscopy for acid-fast bacilli/TB), GeneXpert. There is no CSF microscopy.

Financial

Patients pay an admission fee, and pay for all medications and consumables (private pharmacy on-site). The approximate total cost to the family of a young child admitted for 3 days with pneumonia is N12200 (USD$60.1).

Hospital 4 (H4)

H4 is a government hospital in a city of approximately 500,000 population. It provides a full range of medical, basic surgical, obstetric, paediatric and neonatal inpatient and outpatient services. It has a particularly large role in some specialities (e.g. ophthalmology).

H4 is run by a consultant physician (Chief Medical Director). H4 has been badly affected by industrial action, being closed for approximately 10% of the time in the 18 months prior to the project period. During times of industrial action, H4 closes entirely and typically takes some weeks to resume full operational capacity after re-opening.

H4 is a single-story sprawling complex. It has one large Children's ward that is currently taking up half of the Hall, and two separate neonatal wards (inborn and outborn). The original Children's ward is being refurbished (for the past ~7 years) and has fallen into disrepair. Separate buildings house the Emergency Department, offices, adult wards, and outpatient pharmacy.

Patient flow

Children presenting to the hospital will first be taken to the Children's OPD where they will be seen by a Nurse and Doctor and treatment will be started. Those requiring admission will be sent to the ward. After 4pm the OPDs are closed, and children are seen via the Emergency Department (ED).

It receives referrals from private hospitals, PHCs, General Hospitals. Children requiring referral are sent to tertiary hospitals in neighbouring cities. Transport is available in a hospital ambulance, at a cost to patients (N5,000-10,000) – most will arrange private transport.

H4 has 16 beds in the Children's ward (room for more, but beds are being repaired), and 20 cots for Newborns (in outborn and inborn units).

Staffing

There are 5 permanent doctors for Paediatrics (1 Consultant Paediatrician) and one additional visiting Consultant Paediatrician, and 6 rotating doctors (house officers, corps members). Doctors work on call.

There are 33 nurses who work with children. Three nurses have Paediatric training; most nurses have double Nurse-Midwifery training. Nurses work three shifts to provide 24/7 cover (average 2 per ward).

Oxygen and pulse oximetry

Prior to our study intervention, H4 had access to oxygen concentrators and oxygen cylinders (relying mostly on cylinders). There were empty oxygen cylinders in the paediatric areas, and no working oxygen concentrators. Pulse oximetry was not used anywhere in the hospital. Oxygen therapy was being prescribed to many patients based on clinical signs. Oxygen was moderately expensive, costing N2000 (USD$10) per day. There have been previous attempts to rehabilitate the old oxygen pipe lines – but it was an extremely expensive quote. There was sufficient oxygen tubing present, and nasal prong cannula were available and were reused after washing.

H4 had several key influencers take on leadership roles from an early time. The Chief Medical Director was supportive and delegated responsibility. The paediatrician (head of department) took on substantial responsibility from the beginning. The nurse unit managers in both the paediatric and neonatal areas were actively involved from very early on. The paediatric department formed multi-

disciplinary oxygen team that meet regularly to review progress and challenges with pulse oximetry adoption, a development that served as model for other departments in the hospital.

Systems

The mains power supply (NEPA) is very poor, and it is common to go for weeks without receiving any power. Average supply is 6 hours per week. The hospital relies mostly on generators.

There is a Maintenance team on site, who work mostly with the generators and buildings. Repairs for medical equipment are using a private contractor. There is no preventive maintenance for the concentrators (or any other medical equipment).

H4 had reasonable clinical records but poor administrative records systems.

H4 has regular educational sessions, including Paediatric teaching every morning. There is weekly nursing teaching. Mortality and Morbidity (M&M) meetings are monthly. No standard treatment guidelines are used in paediatrics.

Pharmaceutical supplies are well stocked, using a user-pay system (including essential antibiotics, vaccines, IV fluids, and injection equipment). Essential supplies were available (including bedside glucometer, thermometer, neonatal and adult scales). Infection control equipment was available on every ward (including wash basin, soap, sharps and waste disposal, sterile gloves, chlorhex/iodine, alcohol swabs). Cord clamps and Gentian Violet were not used (use cord in alcohol prepared by nurse), and a working Resuscitaire with neonatal resuscitation equipment (but no neonatal mask).

X-ray facilities are available. Laboratory facilities are available (including Hb, blood group and cross match, urinalysis, smear microscopy for acid-fast bacilli/TB), there is no CSF microscopy.

Financial

Cost of admission is FREE for children under 5 years of age, but patients occasionally pay for cards (N200) or admission folder (N1500) when the hospital runs out of stationary. Patients pay for drugs and consumables at health facility, and if they are not available, patients have to buy from the main pharmacy (external). The approximate total cost to the family of a young child admitted for 3 days with pneumonia is N16500 (USD$82.5).

Hospital 5 (H5)

H5 is a government hospital, exclusively for paediatric care, in a large city of over 3 million population. It provides inpatient and outpatient paediatric services, including nutrition, immunisation, and family planning (no surgical services).

H5 is run by a consultant paediatrician (Chief Medical Director). H5 has been badly affected by industrial action, being closed for approximately 30% of the time in the 18 months prior to the project period. During times of industrial action, H5 closes entirely and typically takes some weeks to resume full operational capacity after re-opening.

H5 is situated in a rambling mansion, with multiple small wards for children of varying ages, and two adjacent neonatal areas. Despite not having any births on-site, H5 still admits quite a few neonates. The OPD and triage area is on the ground floor around the rear of the building. A Nutrition office is nearby, with a covered area for doing teaching. Offices are in a separate building, with the family planning clinic and laboratory situated nearby.

Patient flow

Children presenting to the hospital will first be taken to the Children's OPD where they will be assessed by a Nurse and a Doctor and management will be initiated. If a child needs admission, they will be transferred to one of the Children's or Neonatal wards. The Children's OPD is open 24/7.

H5 receives referrals from all over the state (and beyond). Children requiring referral from H5 are generally sent to the nearby tertiary hospital or a mission hospital. Patients are required to arrange their own transport by private car or taxi.

H5 has 44 beds in the four Children's wards (young children, older children/measles/isolation) and 16 beds for Newborns.

Staffing

In the whole hospital there are 10 permanent doctors (1 Paediatric Consultant), 3 house officers, 4 corps members, 62 nurses, 7 public health nurses, 1 community health extension worker and 7 health assistants.

Doctors work on call, with 3 doctors available after hours. No nurses have Paediatric training; most nurses have double Nurse-Midwifery training. Nurses work three shifts to provide 24/7 cover (3+, 1-2, 1) — 3 or more in the morning, 1-2 in the afternoon, and 1 at night.

Oxygen and pulse oximetry

Prior to our study intervention, H5 had access to oxygen cylinders (but not oxygen concentrators). Pulse oximeters were present but not being used. Oxygen therapy was prescribed to some patients based on clinical signs. Oxygen was moderately expensive, costing N3500 (USD$17.5) per patient. Children are occasionally transferred out because of inadequate oxygen supplies to meet demand. There was sufficient oxygen tubing present, and nasal prong cannulas were used - but the neonatal size nasal prong cannula were difficult to get supply. Nasal prongs are washed and reused.

H5 had several key influencers take on leadership roles from an early time. The paediatrician (medical director) took on substantial responsibility from the beginning. The nurse unit managers in both the paediatric and neonatal areas were involved from very early on, but did not engage until

the full oxygen training. H5 developed an effective multi-disciplinary oxygen team chaired by a senior medical officer that met regularly.

Systems

The mains power supply (NEPA) is very poor, and it is common to go for weeks without receiving any power. Usually it provides only a few hours during the day, and half the night. The hospital relies mostly on generators. The large diesel generator is not used every day, but can provide the entire hospital with power, and is used for up to 7 hours per day (10am-2pm, 7pm-10pm). At other smaller petrol generators are used to provide power to specific areas (e.g. wards, lab, admin).

There is a Maintenance team. Repairs for medical equipment is done by the on-site maintenance team; they maintain and repair medical equipment within their capacity. There is no preventive maintenance for medical equipment.

Individual 'Case Notes' are kept for every admission, with a unique patient identifying number. Documentation is reasonable, including: history/exam/diagnosis/plan, regular nursing observations, treatment prescribed, etc. Sp02 is not reliably recorded and is not routinely done on admission.

H5 had reasonable clinical records and used State Government templates and collated information into the Medical Record Keeping System (HMIS register) based on Case Notes on discharge.

H5 has regular educational sessions, including weekly Grand Rounds for the whole hospital – with presentations from different departments. There is regular nursing education. Mortality and Morbidity (M&M) meetings are held rarely – they used to be regular but it has been curtailed by the current shortage.

No standard treatment guidelines are used in paediatrics. Clinical care depends on prior learning and some doctors have paediatric handbooks. One wall chart describing the use of artemisin for severe malaria was present (drug company wallchart).

Pharmaceutical supplies are well stocked at an on-site pharmacy, using a user-pay system (including essential antibiotics, vaccines, IV fluids, and injection equipment). Essential supplies were available (including bedside glucometer, thermometer, neonatal and adult scales). Infection control equipment was available on every ward (including wash basin, soap, sharps and waste disposal, sterile gloves, chlorhex/iodine, alcohol swabs). Cord clamps and Gentian Violet were not used – staff used cord dipped in alcohol.

X-ray facilities are present – however the machine is faulty and there is no radiographer or radiologist. Laboratory facilities are available (including Hb, blood group and cross match, urinalysis,) – but not CSF microscopy or smear microscopy for acid-fast bacilli/TB.

Financial

Cost of admission is FREE. Patients pay for all medications and consumables. The approximate total cost to the family of a young child admitted for 3 days with pneumonia is N8,500 (USD$42.5).

Hospital 6 (H6)

H6 is a small long-established mission hospital in a city of approximately 500,000 population. It provides a broad range of inpatient and outpatient paediatric, maternity services, surgical and medical services.

H6 is run by an experienced surgeon (Chief Medical Director), and has not recorded any periods of facility closure or industrial action.

H6 is a large single-story compound of multiple connected wings. The Children's ward is in a dedicated building, and contains a section within it for Neonates. The other administrative and clinical buildings stretch over the broad, green grounds, with residences for hospital staff on site.

Patient flow

Children presenting to the hospital will first be taken to the Emergency Department (combined adult and child) where they will be assessed by a Nurse and a Doctor and management will be initiated. If a child needs admission, they will be transferred to the Children's ward.

H6 receives referrals from all over the state, including the tertiary hospitals when they go on strike. Children requiring referral are generally sent to the nearby tertiary hospital. Ambulance services are available and patients pay depending on the distance and urgency.

H6 has 15 inpatient beds on the Children's ward and 5 cots for newborns.

Staffing

In the whole hospital there are 14 permanent doctors, 5 house officer, 50 nurses, 10 midwives, 1 community health officer, 2 community health extension workers, and 25 health assistants.

For Paediatrics and Newborn, there are 3 doctors (1 Consultant Paediatrician), 2 house officers, and 7 nurses (plus some locum nurses as needed. Two nurses have special paediatric training.

Doctors work on call (3 doctors for whole hospital). Nurses work three shifts to provide 24/7 cover. Staff live on the hospital ground and will come to give additional assistance as required.

Oxygen and pulse oximetry

Prior to our study intervention, H6 had access to oxygen concentrators and oxygen cylinders. In the whole hospital there were 8 concentrators, and 12 concentrators had recently been discarded due to defects. However, there were no oxygen cylinders in the paediatric areas and only a single oxygen concentrator was producing medical grade oxygen. Pulse oximetry were present and H6 was the only hospital routinely performing pulse oximetry on all admitted children and newborns (using a fingertip pulse oximeter). Oxygen therapy was prescribed to patients but oxygen was prohibitively expensive, N400 per hour (USD$60 per day).

There is a Maintenance team but do not repair medical equipment. There is no preventive maintenance for medical equipment; only repair on damage. Private technicians repair medical equipment. There was not sufficient oxygen tubing present, and there were limited supply of nasal prong cannulas. Nasal prongs were reused, washing with antiseptic then allowing to dry.

H6 had several key influencers take on leadership roles from an early time. Initially, the hospital paediatrician was the focal person, but she had minimal influence as she was not part of the particular faith community. Later the Director of Nursing Services, a member of hospital

management team, took up the leadership responsibility and served as the chairman of local multi-disciplinary oxygen team. The team meet every month to discuss pulse oximetry and oxygen related care services.

Systems

The mains power supply (NEPA) is fair, providing on average ~8 hours supply per day, but may be absent for some days. The hospital relies on generators. The largest can power the entire hospital and residence quarters.

H6 had reasonable clinical records and administrative records systems and House officers do a quarterly report including the collation of statistics – but these are not archived. Medical Records staff keep basic admission statistics (but not diagnoses).

H6 has regular education sessions. These include morning reviews daily, twice weekly general hospital seminars (with different topics from different departments). There is continuing education for nurses on a quarterly basis. Mortality and Morbidity (M&M) meetings are held regularly (quarterly), in paediatrics.

There are no standard treatment guidelines routinely used in paediatrics.

Pharmaceutical supplies are well stocked at an on-site pharmacy, which patients buy (including essential antibiotics, vaccines, IV fluids, and injection equipment). Essential supplies were available (including bedside glucometer, thermometer, neonatal and adult scales). Infection control equipment was available on every ward (including wash basin, soap, sharps and waste disposal, sterile gloves, chlorhex/iodine, alcohol swabs, cord clamps and GV). Bag and mask were available, in labour ward and on the paediatric ward (with appropriate sized masks).

X-ray facilities are available. Laboratory facilities are available (including Hb, blood group and cross match, urinalysis, CSF microscopy, and smear microscopy for acid-fast bacilli/TB).

Financial

Patients pay a daily admission fee, and pay for all medications and consumables. The approximate total cost to the family of a young child admitted for 5 days with pneumonia is N26450 (USD$132).

Hospital 7 (H7)

H4 is a government hospital in a city of approximately 500,000 population, dedicated to maternal and child health care. It was established 6 years ago as a 'pet project' of the Governor, and as such, it is unique in offering completely free care and having relatively new facilities. It provides inpatient and outpatient paediatric and maternity services, including nutrition, immunisation, and family planning (no surgical services).

H4 is run by an experienced surgeon, and paediatric care is under an experienced paediatrician. H4 has been badly affected by industrial action, being closed for approximately 10% of the time in the 18 months prior to the project period. During times of industrial action, H4 closes entirely and typically takes some weeks to resume full operational capacity after re-opening.

H7 is a large single-story compound of multiple connected wings. The Children's ward and outborn Neonatal ward are situated together, while the inborn Neonatal ward is situated in a different location alongside the Postnatal ward. Medical records and all the office buildings are co-located. The Emergency ward is at the front of the hospital (for both mothers and children).

Patient flow

Children presenting to the hospital will first be taken to the Emergency Department (combined mother and child) where they will be assessed by a Nurse and a Doctor and management will be initiated. If a child needs admission, they will be transferred to one of the Children's or Neonatal wards.

H7 receives referrals from all over the state, and many other states (from private hospitals, PHCs, other state and general hospitals). Many people come from very far (e.g. Lagos) because they know that all the care provided is free of charge. Children requiring referral are generally sent to the nearby tertiary hospital. Ambulance services are available and are free within the state, but patients pay for longer transport.

H7 has 20 inpatient beds on the Children's ward and 28 cots for newborns (15 in the outborn area adjacent to the Children's ward, and 13 in the inborn area beside the Postnatal ward).

Staffing

In the whole hospital there are 17 permanent doctors, 7 house officer, 8 corps members, 220 nurses, and ~30 health assistants.

For Paediatrics and Newborn, there are 8 doctors (1 Consultant Paediatrician), 8 house officers/corps members, and 26 nurses.

Doctors work on call (3 doctors for Paeds, plus 3 for O&G). No nurses have Paediatric training; all nurses have double Nurse-Midwifery training. Nurses work three shifts to provide 24/7 cover.

Oxygen and pulse oximetry

Prior to the project intervention, H7 primarily used piped oxygen. They had two broken concentrators, and rarely use small cylinders. Piped oxygen is provided from very large cylinders ("Bonusses"). It is frequently not available for 3+ days at a time. Many of the outlets throughout the hospital are not actually connected.

In Paediatrics/Neonates there was piped oxygen available at a single outlet in the outborn Neonatal ward and at 3 outlets in the inborn Neonatal ward. There was a single oxygen concentrator on the inborn Neonatal ward which did not produce medical oxygen.

There were no pulse oximeters in the entire hospital. There was not sufficient oxygen tubing present, and there were no nasal prong cannulas. Oxygen was routinely administered using NG tubes taped to the inside of the nares. Oxygen therapy was frequently prescribed. Oxygen (and all care) is provided for free. Children are frequently transferred to the Tertiary hospital because of inadequate oxygen supplies to meet demand.

H7 struggled to establish an effective oxygen team initially, with a single paediatrician providing leadership but encountering significant push-back from particular senior nursing staff. Multidisciplinary ownership improved following full oxygen training with multiple senior nurses stepping up to take responsibility.

Systems

There is a Maintenance team onsite that does electrical work. Repairs for medical equipment is by an external contractor. There is no preventive maintenance for medical equipment.

The mains power supply (NEPA) is poor, providing on average ~4 hours supply per day, and may be absent for entire days. The hospital relies mostly on generators. There are two large diesel generators which can provide the entire hospital with power, and are used for ~16 hours per day.

Record keeping was reasonable, including a computer record system used to collate data by Medical Records staff and electronic scanning of old files (>2 years).

H7 has regular education sessions. These include weekly seminars for Paediatrics and a Teaching ward round three times per week (led by the Consultant Paediatrician). There used to be regular hospital Grand Round presentations, but this has ceased due to lack of money for refreshments. There is occasional nursing education. Mortality and Morbidity (M&M) meetings are no longer held regularly (previously monthly), as there has been no funding for refreshments.

Standard treatment guidelines have been developed by the Consultant Paediatrician for use in paediatrics. These include guidelines on neonatal jaundice, tetanus, malnutrition etc. These have been distributed in booklet form to doctors, and are available on the ward as ward charts (only a few). They have been effectively used for task-shifting, so that nurses can initiate care according to the protocols.

Pharmaceutical supplies are well stocked at an on-site pharmacy, and everything is provided free of charge (including essential antibiotics, vaccines, IV fluids, and injection equipment). Essential supplies were available (including bedside glucometer, thermometer, neonatal and adult scales). There were not enough scales, strips for the glucometer were difficult to obtain supply. Infection control equipment was available on every ward (including wash basin, soap, sharps and waste disposal, sterile gloves, chlorhex/iodine, alcohol swabs). Cord clamps and Gentian Violet are used. Bag and mask were available, but not a full Resuscitaire.

X-ray facilities are not present – patients would be referred to the State Specialist Hospital (there are 4 Xray technicians being paid to do nothing because there is no equipment). Laboratory facilities are available (including Hb, blood group and cross match, urinalysis,) – but not CSF microscopy or smear microscopy for acid-fast bacilli/TB.

Financial

All care is provided free of charge – including no admission fees, medications and supplies, oxygen etc. This is one reason the hospital attracts clients from such a wide catchment area. The hospital is funded by the State government using results-based financing (i.e. paid per package delivered, e.g. normal delivery, C-section etc.) – but payments are almost 12 months late so the hospital owes millions. This has led to user-fees being introduced (unofficially) during the past couple of years.

Hospital 8 (H8)

H8 is a government hospital, in a large city of over 3 million population, primarily for maternity services. It is a teaching hospital for obstetrics, and provides paediatric and neonatal inpatient services alongside.

H8 is run by a consultant obstetrician-gynaecologist. H8 has been badly affected by industrial action, being closed for approximately 30% of the time in the 18 months prior to the project period. During times of industrial action, it closes entirely and typically takes some weeks to resume full operational capacity after re-opening.

H8 has one large Children's ward in a dedicated building, separate Inborn and Outborn wards on second floor, a dedicated Children's OPD area, and multiple other buildings for maternity services, ED, theatre, offices, pharmacy etc.

Patient flow

Children presenting to the hospital will first be taken to the Children's OPD where they will be assessed by a Nurse and a Doctor and management will be initiated. If a child needs admission, they will be transferred to the Children's ward (or SCBU/Neonatal ward if <28 days old). The Children's OPD closes at 2pm. After this time children present to the Emergency Department (ED).

H8 serves a catchment area covering the whole city and the surrounding areas of H12 and neighbouring states. As a major Maternity hospital, H8 receives referrals from private hospitals, PHCs, and other government and mission hospitals.

Children requiring referral from H8 are sent to nearby tertiary or large mission hospitals. Transport is available in a hospital ambulance at the patient's expense, however patients usually arrange a vehicle privately as that is more affordable.

H8 has 22 beds in the Children's ward, and 24 beds for Newborns.

Staffing

In the whole hospital there are 40 permanent doctors, 7 house officers, 3 corps members, 202 nurses, 8 public health nurses, 4 community health officers, 4 community health extension workers, and 55 health assistants.

In Paediatrics and neonates there are 9 permanent doctors plus 1 Consultant Paediatrician (mostly administrative), and 2 rotating doctors (i.e. HMO, Corps members). Doctors work on call, with 1-2 to cover the Children's ward and SCBU.

There are 31 nurses who work with children and newborns, including 15 in the SCBU and 16 on the Children's wards. No nurses have Padiatric training and all rotate to other wards; all nurses have double Nurse-Midwifery training. Nurses work three shifts to provide 24/7 cover.

Oxygen and pulse oximetry

Prior the project intervention, H8 used mostly oxygen cylinders (there was a single concentrator in theatre). Newborns are charge N1000 per hour for oxygen and older children (and adults) are charged N2000 per hour (USD$120-240 per day). This cost if frequently unaffordable and many patients are unable to pay their bills. The funds are reportedly used to offset other hospital expenses. If oxygen was unaffordable to patients staff reported that they would provide it anyway.

H8 had no pulse oximeters in the entire hospital. There was sufficient oxygen tubing present. The SCBU had nasal prongs available which may be reused after washing. The Children's ward at a small face mask available.

H8 struggled to establish an effective oxygen team initially, with leadership being provided by a Senior Medical Officer and who serves as the Head of Paediatric Department, with little support from the hospital management team. H8 formed effective oxygen team after full oxygen system training and increased interest from the State Hospital Management Board.

Systems

The mains power supply (NEPA) is very poor, and it is common to go for entire weeks without receiving any power. The hospital relies mostly on generators. Diesel generators can provide the entire hospital with power, and are used for 7 hours per day (10am-2pm, 7pm-10pm). At other times smaller generators are used to provide power to select areas (e.g. theatre, lab, ED, maternity, SCBU).

There is an onsite Maintenance team who look after the generators, power, buildings etc. Repairs for medical equipment is done by the hospital technicians, there is no routine preventive maintenance.

Record keeping is reasonable, but summary statistics are limited – despite using the State Government template – and staff report that the recorded diagnosis is often missing or inaccurate. The Medical Records staff were very keen for assistance in improving medical records, pointing out the challenges with their current system.

H8 has regular educational sessions, including a clinical presentation very week, and monthly presentations specifically for nurses. H8 is a training site for Obstetrics/Gynaecology. Mortality and Morbidity (M&M) meetings are held once per month.

No standard treatment guidelines are used in paediatrics. Part of a WHO guideline for the treatment of malaria was pasted on the wall of the Children's ward.

Pharmaceutical supplies are well stocked, using a user-pay system (including essential antibiotics, vaccines, IV fluids, and injection equipment). There are three pharmacies on-site, using a Private-public partnership system. Essential supplies were available (including bedside glucometer, thermometer, neonatal and adult scales), using a user-pay system. Infection control equipment was available on every ward (including wash basin, soap, sharps and waste disposal, sterile gloves, chlorhex/iodine, alcohol swabs). Cord clamps and Gentian Violet were available in Birth Suite, and a working Resuscitaire and neonatal resuscitation equipment was reportedly available.

X-ray facilities are available. Laboratory facilities are available (including Hb, blood group and cross match, urinalysis, CSF microscopy, smear microscopy for acid-fast bacilli/TB).

Financial

Cost of admission is FREE, but patients pay for all medications and consumables. The approximate total cost to the family of a young child admitted for 3 days with pneumonia is N60500 (USD$302.5).

Hospital 9 (H9)

H9 is a government hospital in a city with population less than 500,000. It is one of three hospitals in the area – the others being a Christian mission hospital, and the Muslim hospital. It was elevated to from General to State Hospital status prior to the project period, but had not had significant upgrades. It has received some support from the local community, including the staff themselves, and some NGOs to do upgrades such as the pathology unit.

H9 is run by a senior medical consultant, who attends the hospital just a few days each week (lives far away) and was eager to transfer elsewhere. H9 has been badly affected by industrial action, being closed for approximately 30% of the time in the 18 months prior to the project period. During times of industrial action, it closes entirely and typically takes some weeks to resume full operational capacity after re-opening.

H9 is a single-story complex. It has one room for Children, as part of the General wing. Newborns are generally cared for in Maternity with their mothers, but there are multiple small rooms used for Maternity overflow, and one that is used for preterm and sick newborns. The hospital was doing extensions at the end of the Maternity ward. Separate buildings house the lab, offices, and other offices and the OPD/triage area.

Patient flow

Children presenting to the hospital will first be taken to the main OPD/triage/registration area at the front of the hospital. Here they are seen (together with adults) then transferred to the Children's ward if they need admission. This is the same during and after hours.

H9 serves a catchment area covering the region, including across state borders. Children requiring referral are sent to the local mission hospital or tertiary hospitals in other cities. Transport is by private transport, there is no Ambulance available.

H9 has 9 beds/cots in the Children's ward, and 4 cots for Newborns (in a small room adjacent to Maternity ward).

Staffing

For the whole hospital there are 6 permanent doctors, no house officers, no corps members, 47 nurses, and 19 health assistants.

For the General wing (Adult and Children's Medical) there are 6 permanent doctors (including 1 Consultant Family Physician), no house officers, 1 corps members. Doctors work on call, with 1 doctor covering the entire hospital after hours.

There are 11 nurses on the General wing, plus additional in Midwifery who work with newborns. No nurses have Paediatric training; most nurses have double Nurse-Midwifery training. Nurses work three shifts to provide 24/7 cover for the General wing.

Oxygen and pulse oximetry

Prior to our project intervention, H9 had only one medium oxygen cylinder for the entire hospital. It was an old cylinder which was suspected to have a leak. Oxygen is rarely used for patients, and when it is patients are charged N5,000-10,000 (USD$25-50).

H9 has no pulse oximeters in the entire hospital. There was inadequate oxygen tubing present, and only adult face masks were available.

H9 had no key influencers to champion pulse oximetry for a long time, largely due to a combination of new/rotating staff and internal staff disagreements between the senior medical officer and nurse. With frequent supervisory visits, the coordination team identified a different senior nursing officer who championed the campaign for pulse oximetry. Later the hospital secretary filled the leadership vacuum and coordinated activities of the oxygen team. H9 finally had one of the best local multi-disciplinary oxygen teams.

Systems

The mains power supply (NEPA) is very poor, and it provides at best a few hours each day. The hospital relies mostly on generators. The large diesel generator (30KV) is used to provide power for 2-4 hours overnight. Small generators are used for specific purposes during the day (e.g. theatre).

There is a Maintenance team on site, who work mostly with the generators and buildings. Repairs for medical equipment are using a private contractor. There is no preventive maintenance any medical equipment.

Record keeping is reasonable, but there is little use of summary statistics.

H9 has some educational sessions. Ward rounds and Clinical meetings for the whole hospital. Mortality and Morbidity (M&M) meetings are not held. No standard treatment guidelines are used in paediatrics.

Pharmaceutical supplies are well stocked, using a user-pay system (including essential antibiotics, vaccines, IV fluids, and injection equipment). Essential supplies were available (including bedside glucometer, thermometer, neonatal and adult scales). Infection control equipment was available on every ward (including wash basin, soap, sharps and waste disposal, sterile gloves, chlorhex/iodine, alcohol swabs). Cord clamps and Gentian Violet were used, but there was no newborn resuscitation equipment available.

X-ray facilities are NOT available. Laboratory facilities are available (including Hb, blood group and cross match, urinalysis, smear microscopy for acid-fast bacilli/TB, CSF microscopy). The lab also does GeneXpert testing, blood and CSF cultures, and biochemistry. The lab facilities are quite impressive, with substantial support from NGOs, staff and the local community.

Financial

Cost of admission is FREE. Patients pay for all medications and consumables. The approximate total cost to the family of a young child admitted for 5 days with pneumonia is N12060 (USD$60.3).

Hospital 10 (H10)

H10 is a large Catholic Mission hospital, in a city of over 3 million population. It provides a full range of medical, surgical, obstetric, paediatric and neonatal inpatient and outpatient services, including immunisation.

H10 is run by a non-medical Hospital Administrator, and experienced Family Medicine Consultant as Medical Director. It has not recorded any periods of facility closure or industrial action. During times of industrial action in nearby government hospitals, the number of paediatric admissions to H10 has been noted to increase substantially.

H10 has one large Children's ward (partially divided into two sections), a separate Isolation ward, a 'SCBU' for sick newborns, and a general Neonatal ward mostly for phototherapy. These are all situated in the same long building (together with the Medical Director's office and the crèche). A separate building houses the Emergency Department and Children's Outpatient Department (OPD), and outpatient pharmacy, behind which the Maintenance building and 2 diesel generators are housed. A separate multi-story building houses the inpatient pharmacy, theatre, private ward, and other wards.

Patient flow

Children presenting to the hospital will first be taken to the Children's OPD where they will be assessed by a Nurse and a Doctor and management will be initiated. If a child needs admission, they will be transferred to the Children's ward (or general Neonatal ward if <28 days old). The Children's OPD closes at 2pm. After this time children present to the Emergency Department (ED).

H10 receives referrals from private hospitals, other mission hospitals, PHCs, and from more distant private and Catholic hospitals, particularly its sister hospitals. Children requiring referral from H10 are sent to the nearby tertiary hospital. Transport is available in a hospital ambulance and the patient's expense.

H10 has 38 beds in the Children's ward, and 25 beds for Newborns (15 for post-C-section newborns in the SCBU and 10 in the general Neonatal ward for inborn and outborn babies).

Staffing

In the whole hospital there are 34 permanent doctors, 84 nurses, and >100 health assistants. There are additional part-time Consultants who visit regularly.

There are 6 permanent doctors working in Paediatrics, all who are Family Medicine trainees. There are no rotating doctors (i.e. HMO, Corps members). There is one part-time Consultant Paediatrician who visits twice per week. Doctors work on call.

There are 18 nurses who work with children and newborns, including 7 in the SCBU and 11 on the Children's and general Neonatal wards. No nurses have Paediatric training; all nurses have double Nurse-Midwifery training. Nurses work three shifts to provide 24/7 cover.

Oxygen and pulse oximetry

Prior to the project intervention, H10 used both oxygen cylinders and oxygen concentrators. However, the concentrators were not producing medical oxygen. Patients are charged N800 per hour for oxygen (USD$96 per day), irrespective of whether using the concentrator or cylinder. Children are frequently transferred out because the family cannot afford to pay for oxygen at H10

(approximately on a weekly basis). H10 had no pulse oximeters in the entire hospital (except in the integrated observation machines in theatre). There was sufficient oxygen tubing present, but only the neonatal and adult size nasal prong cannula were available. Nasal prong cannulas are washed and reused (at no cost to the patient).

The medical director was an early and eager advocate for pulse oximetry and formed a multi-disciplinary oxygen team 3 months after introducing pulse oximetry in response to feedback from the project coordination team. The team included the most senior staff (Administrator, Chief Medical Director, Matron, senior Technician, Accountant, medical record officer). The first meeting was instrumental in formally adopting pulse oximetry as part of vital signs for all inpatients.

Systems

The mains power supply (NEPA) is very poor, and it is common to go for weeks without receiving any power. The hospital relies mostly on generators. The large diesel generator can provide the entire hospital with power, and is used for 7 hours per day (10am-2pm, 7pm-10pm). At other times a smaller diesel generator (18KVA) is used to provide power to select areas (e.g. theatre, lab, ED, maternity, SCBU). Solar power was previously available through two sets of ground-mounted PV panels (~24 panels) – but now broken.

There is a Maintenance team on site, who work mostly with the generators and buildings. Repairs for medical equipment is done by a private technician who is called in. There is no preventive maintenance for the concentrators (or any other medical equipment).

Record keeping is relatively good, and the Medical Director compiles regular reports based on the Admission books from each ward. Medical Records staff do not collate information or produce reports.

H10 has regular educational sessions, including compulsory continuing medical education (CME) for resident doctors. It is a training site for Family Medicine. Pharmaceutical companies conduct drug presentations twice per month. There is regular nursing education. Mortality and Morbidity (M&M) meetings are held once per month. No standard treatment guidelines are used in paediatrics.

Pharmaceutical supplies are well stocked, using a user-pay system (including essential antibiotics, vaccines, IV fluids, and injection equipment). Essential supplies were available (including bedside glucometer, thermometer, neonatal and adult scales). Infection control equipment was available on every ward (including wash basin, soap, sharps and waste disposal, sterile gloves, chlorhex/iodine, alcohol swabs). Cord clamps and Gentian Violet were available in Birth Suite, and a working Resuscitaire and neonatal resuscitation equipment was reportedly available.

X-ray facilities are available. Laboratory facilities are available (including Hb, blood group and cross match, urinalysis, CSF microscopy, smear microscopy for acid-fast bacilli/TB). The lab also does CSF and blood culture, and biochemistry.

Financial

Patients pay a daily admission fee, and pay for all medications and consumables. Inability to afford care at H10 is frequently a reason for transfer. The approximate total cost to the family of a young child admitted for 5 days with pneumonia is N37800 (USD$189).

Hospital 11 (H11)

H11 is a Catholic mission hospital, located in a city of <500,000 population. It provides a full range of medical, basic surgical, obstetric, paediatric and neonatal inpatient and outpatient services.

H11 is run by Hospital Administrator (Matron) who is of Catholic background. H11 has a Chief Medical Director, who advises on medical related issues but he is answerable to the Hospital Administrator. It has not recorded any periods of facility closure or industrial action.

H11 is a mostly single-story complex in a crowded urban area. The paediatric ward sits alongside a dedicated ward for abandoned/orphaned children (aka orphanage).

Patient flow

Depending on severity, children presenting to the hospital either go to the OPD or Emergency Department (ED) where they will be seen by a Doctor and treatment will be started. Those requiring admission from the OPD will be sent to the ward. In ED they are either admitted at the Emergency or sent to the ward. After 4pm the OPDs are closed, and children are seen via the Emergency Department (ED). In-borns are admitted via the maternity while outborns are admitted through the ED.

H11 serves the local city area. It receives referrals from PHCs, other mission and government and private hospitals, and some tertiary hospitals. Children requiring referral are sent to the regional Teaching Hospital. Transport is available in a hospital ambulance, at a cost.

H11 has 12 beds in the Children's ward (including 4 isolation), and 2 cots for Newborns.

Staffing

In the whole hospital there are 6 permanent doctors, 1 house officer, 4 Corps members, 18 nurses, 15 midwives, 12 community health extension workers, and 8 health assistants.

There is one doctor for paediatric areas. Two doctors are on call for the whole hospital after hours.

Four nurses work with children and newborns. No nurses have Paediatric training. Nurses work three shifts to provide 24/7 cover.

Oxygen and pulse oximetry

Prior to the project intervention, H11 mostly used oxygen cylinders but did also have a couple of concentrators (non- functional).

H11 had one pulse oximeter in the paediatric area, and this was being sporadically used.

Oxygen was infrequently prescribed for children, but was prohibitively expensive, N6000 per cylinder (USD$30).

Systems

Power supply from mains was limited, and the hospital relied mostly on generators. The large diesel generator (100KVA) can provide the entire hospital with power, and is used for most of the day. Solar power is available at the Antenatal department.

Repairs for medical equipment are using a private contractor. There is no preventive maintenance for the concentrators (or any other medical equipment).

Record keeping was quite good, and included computer-based summary data for each ward.

H11 has regular educational sessions, including monthly clinical meetings and monthly drug detailing meetings. Mortality and Morbidity (M&M) meetings are not held. No standard treatment guidelines are used in paediatrics.

Pharmaceutical supplies are well stocked, using a user-pay system (including essential antibiotics, vaccines, IV fluids, and injection equipment). Essential supplies were available (including bedside glucometer, thermometer, neonatal and adult scales).

Infection control equipment was available on every ward (including wash basin, soap, sharps and waste disposal, sterile gloves, chlorhex/iodine, alcohol swabs). Cord clamps and Gentian Violet were not used (use cord in alcohol prepared by nurse), and a working Resuscitaire with neonatal resuscitation equipment was reported to be present.

X-ray facilities are available. Laboratory facilities are available (including Hb, blood group and cross match, urinalysis, smear microscopy for acid-fast bacilli/TB), and CSF microscopy. The lab does not do GeneXpert testing; samples taken are sent to nearby government hospital for processing.

Financial

Patients pay daily admission fees and pay for all medications and consumables (public-private partnership). The approximate total cost to the family of a young child admitted for 3 days with pneumonia is N35480 (USD$177.4).

Hospital 12 (H12)

H12 is a small government hospital, in a city of <500,000 population. It provides a full range of medical, basic surgical, obstetric, paediatric and neonatal inpatient and outpatient services.

H12 is run by a family medicine consultant. H12 has been badly affected by industrial action, being closed for approximately 30% of the time in the 18 months prior to the project period. During times of industrial action, it closes entirely and typically takes some weeks to resume full operational capacity after re-opening.

H12 is a single-story sprawling complex. It has one large Children's ward, and a smaller isolation ward, which is located alongside the Paediatric OPD area. Newborns are cared for within the Maternity ward without a dedicated area. A separate building houses the Emergency Department, offices, adult wards, and outpatient pharmacy. The Maintenance building is way down the back, and the 2 diesel generators are on lawns between wards.

Patient flow

Children presenting to the hospital will first be taken to the general OPD where they will be triaged by a Nurse and referred either to the Children's ward or the Children's OPD. After 4pm the OPDs are closed, and children are seen via the Emergency Department (ED).

H12 receives referrals from private hospitals, PHCs, General Hospitals in the region. Children requiring referral are sent to tertiary hospitals in neighbouring cities. Transport is available in a hospital ambulance, and it is usually free (unless there are fuel scarcity or the hospital lacks funds).

H12 has 26 beds in the Children's ward, and 10 cots for Newborns (in the main Maternity ward, not separated from well babies or mothers).

Staffing

In the whole hospital there are 8 permanent doctors (2 Consultants in Family Med and O&G), 2 house officers, 5 corps members, 83 nurses, and 34 health assistants.

There is a single permanent doctor in Paediatrics, and 6 rotating doctors (3 HMO, 3 Corps members). There is one part-time Consultant Paediatrician who visits twice per week. Doctors work on call, with 2 doctors covering the entire hospital after hours.

There are 11 nurses who work with children, and 15 on the maternity ward, who rotate wards every 1-2 years. No nurses have Paediatric training; all nurses have double Nurse-Midwifery training. Nurses work three shifts to provide 24/7 cover.

Oxygen and pulse oximetry

Prior to the project intervention, H12 used oxygen cylinders and had no oxygen concentrators. There were no cylinders in the paediatric areas but they would reportedly be moved as required. Patients were charged N1000 per hour for oxygen (USD$120 per day), and it is only used as an interim measure while awaiting transfer out.

H12 has no pulse oximeters in the entire hospital. There was insufficient oxygen tubing present, and only the adult size nasal prong cannula were available. NG tubes are used for older children, taped to the nares. A face mask is used in theatre. Nasal prongs are reused after cleaning with methylated spirit.

H12 had no key influencers to champion pulse oximetry for a long time, due to frequent staff rotation and internal staff disagreements. Later the hospital secretary became actively involved and recruited other clinical staff as key influencers. With increasing oversight functions from the state hospital management board, H12 formed active local multi-disciplinary oxygen team.

Systems

The mains power supply (NEPA) is very poor, and it is common to go for weeks without receiving any power. The hospital relies mostly on generators. The large diesel generators can provide the entire hospital with power, and are used for 8 hours per day (10am-3pm, 7pm-10pm) – but less during a fuel crisis.

There is a Maintenance team on site, who work mostly with the generators and buildings. Repairs for medical equipment are done in Ibadan – equipment gets sent out. There is no preventive maintenance for the concentrators (or any other medical equipment).

Record keeping is reasonable, and Medical Records staff collate information into the State reporting books and the Secretary prepares monthly reports.

H12 does not have regular educational sessions. Occasional sessions are held for doctors (2-3 per year), nurses (every 1-2 months), and including quarterly pharmaceutical company drug presentations. Mortality and Morbidity (M&M) meetings are not held. No standard treatment guidelines are used in paediatrics.

Pharmaceutical supplies are well stocked, using a user-pay system (including essential antibiotics, vaccines, IV fluids, and injection equipment). Essential supplies were available (including bedside glucometer, thermometer, neonatal and adult scales). Infection control equipment was available on every ward (including wash basin, soap, sharps and waste disposal, sterile gloves, chlorhex/iodine, alcohol swabs). Cord clamps and Gentian Violet were available in Birth Suite, and a working Resuscitaire - but neonatal resuscitation equipment was absent.

X-ray facilities are available. Laboratory facilities are available (including Hb, blood group and cross match, urinalysis, smear microscopy for acid-fast bacilli/TB), there is no CSF microscopy. The lab also does GeneXpert testing.

Financial

Patients pay an admission fee and pay for all medications and consumables. The approximate total cost to the family of a young child admitted for 5 days with pneumonia is N27850 (USD$139.25).
