# Supplementary material for: Adoption of paediatric and neonatal pulse oximetry by 12 hospitals in Nigeria: a mixed-methods realist evaluation
Source: BMJ Glob Health. 2018 Jun 26;3(3):e000812. doi: 10.1136/bmjgh-2018-000812 (PMC6035503; doi:10.1136/bmjgh-2018-000812)
Supplement: Supplementary data [file bmjgh-2018-000812supp002.pdf]

Sample of potential program theories derived from Realist Review[1] and other theories of change[2-7]

This table shows some of the broad, diverse program theories that were considered during the initial stage of theory identification, articulated as basic CMO (context-mechanism-outcome) configurations.

Intervention component	Context	Mechanism	Outcome
Staff education…	When there is low knowledge about pulse oximeters, but some clinical expertise…	Improves staff knowledge in using pulse oximetry…	Increasing the use of pulse oximetry in clinical practice.
Practical staff training and supervised practice…	When there is little experience with pulse oximeters, but some general clinical skills…	Improves staff skill and self-efficacy in using pulse oximetry…	Increasing the use of pulse oximetry in clinical practice.
Learner-centred training approaches, and specific training for trainers…	In a learning culture (or potential learning culture)…	Encourages pulse oximetry knowledge and skills to be passed on and developed…	Enhancing adoption and sustainability of pulse oximetry practices.
Learner-centred training approaches, and use of respected staff as ‘champions’…	When there is healthcare worker pride in professional roles, and complementary existing practices…	Links (new) pulse oximetry practices to existing professional roles and existing clinical tasks…	Enhancing adoption and sustainability of pulse oximetry practices.
Simple guidelines, practical learner-centred training, and use of respected staff as ‘champions’…	When there are no existing pulse oximetry guidelines, but respect for protocols…	Enhances healthcare workers’ behavioural control relating to pulse oximetry use…	Enhancing adoption and sustainability of pulse oximetry practices.
Clinical audit activities…	In a culture of reflective practice and learning…	Convinces healthcare workers that pulse oximetry will lead to better patient outcomes (and provides professional recognition)…	Enhancing adoption and sustainability of pulse oximetry practices.
Visible protocols and supportive supervision…	In a culture of family-centred care…	Convinces patients/families that pulse oximetry will lead to better patient outcomes (and provides professional recognition)…	Enhancing adoption and sustainability of pulse oximetry practices.
Feedback on performance (+/- reward or punishment)…	When there is healthcare worker professional pride (and ego)…	Reinforces pulse oximetry adoption…	Increasing the use of pulse oximetry in clinical practice.
Visible protocols…	In a busy work environment…	Reminds healthcare workers to use pulse oximetry routinely…	Increasing the use of pulse oximetry in clinical practice.
Simple guidelines…	When there are no existing pulse oximetry guidelines, but respect for protocols…	Makes it easy for healthcare workers to do pulse oximetry well…	Enhancing adoption and sustainability of pulse oximetry practices.

Supportive supervision and reminders…	In a collegial work environment…	Associates positive feelings with doing pulse oximetry, increasing motivation and intention…	Increasing the use of pulse oximetry in clinical practice.
Provision of good quality, easy-to-use pulse oximeters…	To healthcare workers who are informed and motivated…	Makes it easy for healthcare workers to do pulse oximetry well…	Enhancing adoption and sustainability of pulse oximetry practices.
Provision of oxygen delivery equipment…	To healthcare workers who are informed and motivated…	Motivates healthcare workers to use pulse oximetry and appreciate its benefits…	Increasing the use of pulse oximetry in clinical practice.
Assistance with sustainable financing mechanisms…	In low-resource, user-pay hospitals…	Enables hospitals to sustain equipment and provide pulse oximetry services equitably and affordably to patients…	Enhancing sustainability of pulse oximetry practices.
Identification and support of local 'champions'…	When there is respect for seniors (and peers), and professional pride…	Encourages users to model pulse oximetry use from colleagues…	Increasing the use of pulse oximetry in clinical practice.
Encouraging the formation of multi-disciplinary oxygen teams…	When existing relationships and communication is lacking…	Fosters interdisciplinary cooperation, collaboration, and respect…	Enhancing sustainability of pulse oximetry practices.
Formally endorsed guidelines…	When there is respect for authority and protocols…	Establishes a standard and norm relating to pulse oximetry, and enables enforcement…	Enhancing adoption and sustainability of pulse oximetry practices.
Support for record-keeping and data feedback…	When existing data collection and reporting systems are weak (or non-responsive)…	Empowers clinical leaders and managers to assess and respond to substandard clinical care…	Enhancing adoption and sustainability of pulse oximetry practices.
Sharing of hospital performance between hospitals and to health authorities…	When hospital managers have respect for authority, and pride in hospital performance…	Makes hospital leaders feel accountable, and build pride in their achievements…	Enhancing adoption and sustainability of pulse oximetry practices.
Health authorities provide practical support to hospitals…	When hospitals depend on health authorities and have ways of receiving support…	Helps hospital leaders make the necessary structural changes and mobilise resources…	Enhancing sustainability of pulse oximetry practices.
Practical technical training for engineers/technicians…	When technicians have responsibility for medical equipment…	Enhances technician capability to do preventive and corrective maintenance and repairs…	Enhancing sustainability of pulse oximetry practices.

Practical technical training for engineers/technicians and the formation of multi-disciplinary oxygen teams…	When recognition and respect for the role of technicians is poor…	Enhances recognition and value of the technician's role…	Enhancing sustainability of pulse oximetry practices.
Simple technical protocols, and technical assistance…	When technicians are busy and have limited skills…	Makes pulse oximetry maintenance and repairs easy, and enhances access to technical support…	Enhancing sustainability of pulse oximetry practices.
Team-based training and formation of multi-disciplinary oxygen teams…	When oxygen-related activities are poorly coordinated…	Builds ownership, value and pride in pulse oximetry…	Enhancing sustainability of pulse oximetry practices.
Transparent cost advice and financial records…	When hospitals have experience procuring oxygen equipment, but are being heavily supported by external agencies in this project…	Convinces hospital leaders of cost-benefits, encourages better care of pulse oximeters…	Enhancing sustainability of pulse oximetry practices.
Formation of multi-disciplinary oxygen teams…	Lack of institutional experience with pulse oximetry (and oxygen)…	Builds institutional knowledge about pulse oximeter selection, procurement, maintenance, repair...	Enhancing sustainability of pulse oximetry practices.

References

1. Graham H, Tosif S, Gray A, et al. Providing oxygen to children in hospitals: a realist review. *Bull World Health Organ* 2017;95(4):288-302. doi: 10.2471/blt.16.186676
2. Bandura A. Social foundations of thought and action: A social cognitive theory: Prentice-Hall, Inc 1986.
3. Michie S, Johnston M, Abraham C, et al. Making psychological theory useful for implementing evidence based practice: a consensus approach. *Qual Saf Health Care* 2005;14(1):26-33. doi: 10.1136/qshc.2004.011155
4. Michie S, van Stralen MM, West R. The behaviour change wheel: a new method for characterising and designing behaviour change interventions. *Implement Sci* 2011;6:42. doi: 10.1186/1748-5908-6-42
5. Rogers EM. Diffusion of Innovations. 3rd ed: Collier Macmillan Canada 1983.
6. Wisdom JP, Chor KH, Hoagwood KE, et al. Innovation adoption: a review of theories and constructs. *Adm Policy Ment Health* 2014;41(4):480-502. doi: 10.1007/s10488-013-0486-4
7. By RT. Organisational Change Management: A Critical Review. *Journal of Change Management* 2005;5(4):369-80.
