# Supplementary material for: Adoption of paediatric and neonatal pulse oximetry by 12 hospitals in Nigeria: a mixed-methods realist evaluation
Source: BMJ Glob Health. 2018 Jun 26;3(3):e000812. doi: 10.1136/bmjgh-2018-000812 (PMC6035503; doi:10.1136/bmjgh-2018-000812)
Supplement: Supplementary data [file bmjgh-2018-000812supp003.pdf]

Test 1 – Pulse Oximetry and Oxygen (Learner)   Study ID:____________________

INSTRUCTIONS

Test 1 is designed to test the knowledge and skills of doctors and nurses regarding the clinical use of pulse oximetry and oxygen. Individual results must not be made public (without the subject's consent).

- Please use a black or blue ballpoint pen.
- Print all written entries with BLOCK CAPITAL LETTERS.
- Mark boxes with a cross (X) where requested (e.g. ☒).
- Answer every question.
- If you make an error - draw a line through the error and write the correct value next to it. Date and initial the correction.

**PART A – General information**		
1	Hospital Name	
2	Date of birth (dd/mm/yyyy)	__ __/__ __/__ __ __ __
3	Age	__ __ Years
4	Sex	☐1 Male ☐2 Female
5	Job Title	
6	Job Category	☐1 Nurse<br>☐2 Nurse Assistant<br>☐3 Student Nurse (training)<br>☐4 House Officer / Corps Member<br>☐5 Resident (Family Medicine or Paediatric training)<br>☐6 Consultant Family Physician<br>☐7 Consultant Paediatrician<br>☐8 Other Doctor (specify):____________________<br>☐9 Other Job Category (specify): ______________
7	How long have you worked at this hospital?	__ __ Years __ __ Months
8	How long have you worked in Paediatrics?	__ __ Years __ __ Months
9	How long have you worked in Maternity or Newborn care?	__ __ Years __ __ Months
**PART B - Training on pulse oximetry and the use of oxygen for children**		
10	Did you receive <u>training on pulse oximetry</u> during your Doctor/Nurse training?	☐0 NO ☐1 YES
11	Did you receive <u>training on the use of Oxygen for Children</u> during your Doctor/Nurse training?	☐0 NO ☐1 YES
12	Have you used the "Pulse oximetry Training Video"?<br>(provided by the Oxygen Project Team)	☐0 NO<br>☐1 YES – I used it in the past month<br>☐2 YES – I used it in the past year<br>☐3 YES – I used in more than a year ago
13	Have you attended the "Clinical Use of Oxygen" course?<br>(provided by the Oxygen Project Team)	☐0 NO<br>☐1 YES – I attended in the past month<br>☐2 YES – I attended in the past year<br>☐3 YES – I attended more than a year ago
14	Have you had any other training on Pulse Oximetry or the use of Oxygen for Children <u>at your hospital</u>?	☐0 NO<br>☐1 YES – I attended in the past month<br>☐2 YES – I attended in the past year<br>☐3 YES – I attended more than a year ago<br>*Specify:*
15	Have you had any other training on Pulse Oximetry or the use of Oxygen for Children <u>anywhere else</u>?	☐0 NO<br>☐1 YES – I attended in the past month<br>☐2 YES – I attended in the past year<br>☐3 YES – I attended more than a year ago<br>*Specify:*

PART C – Quiz. Answer True or False for every question.			
	EXAMPLE. Nigeria is:		
	a. A country in Africa	☒1 TRUE	☐2 FALSE
	b. An island in Asia	☐1 TRUE	☒2 FALSE
1	The **pulse oximeter measures**: **YES/NO PULSE OXIMETRY Q's**		
	a. The patient's respiratory rate (RR)	☐1 TRUE	☐2 FALSE
	b. The patient's heart rate (HR)	☐1 TRUE	☐2 FALSE
	c. Percentage of haemoglobin saturated with oxygen	☐1 TRUE	☐2 FALSE
	d. The patient's blood pressure (BP)	☐1 TRUE	☐2 FALSE
2	**False readings on an oximeter** can be caused by:		
	a. Strong lights shining on the probe	☐1 TRUE	☐2 FALSE
	b. Dark skinned patients	☐1 TRUE	☐2 FALSE
	c. Breathing oxygen from a mask	☐1 TRUE	☐2 FALSE
	d. Nail varnish (paint on nails)	☐1 TRUE	☐2 FALSE
3	Regarding **oxygen saturations** (SpO2):		
	a. Saturation should be measured in every child that is admitted to hospital	☐1 TRUE	☐2 FALSE
	b. Saturation of 88% is OK in a healthy 2-year-old	☐1 TRUE	☐2 FALSE
	c. Saturation is normally less than 95% in children	☐1 TRUE	☐2 FALSE
	d. Pre-term neonates normally have lower saturations than older children	☐1 TRUE	☐2 FALSE
4	The following clinical signs indicate the **need for urgent resuscitation**:		
	a. Fever	☐1 TRUE	☐2 FALSE
	b. Severe respiratory distress (e.g. grunting, gasping, severe chest indrawing)	☐1 TRUE	☐2 FALSE
	c. Convulsions (seizures, fits)	☐1 TRUE	☐2 FALSE
	d. Coma (unconscious or barely conscious)	☐1 TRUE	☐2 FALSE
5	**Oxygen concentrators**: **OTHER QUESTIONS – not included in analysis**		
	a. Take a few minutes to reach adequate oxygen concentration	☐1 TRUE	☐2 FALSE
	b. Must be turned off for at least one hour every day	☐1 TRUE	☐2 FALSE
	c. Must have the external filter cleaned every week	☐1 TRUE	☐2 FALSE
	d. Can be used for more than one patient simultaneously	☐1 TRUE	☐2 FALSE
6	To **test whether oxygen is reaching the patient** you should:		
	a. Listen for a noise – any noise is good	☐1 TRUE	☐2 FALSE
	b. Look at the flow meter – it shows what flow is reaching the patient	☐1 TRUE	☐2 FALSE
	d. Put the nasal prongs in water and look for bubbles – bubbles show gas flow through the prongs	☐1 TRUE	☐2 FALSE
	e. Look at the colour of the patient's skin – if they are not blue the oxygen must be working	☐1 TRUE	☐2 FALSE

PART D – Scenarios. Choose the best action for each of the following scenarios.

	EXAMPLE SpO2(%) PR(bpm)<br>99 117<br>Pleth<br>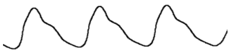	EXAMPLE: A 4-year-old child has a cough and fever.<br>☒1 This is a normal reading, continue routine care, this is a normal reading<br>☐2 The SpO2 is low, start oxygen therapy<br>☐3 Do nothing, check again in 10 minutes<br>☐4 Poor pleth waveform, check the probe placement and try again<br>☐5 This is an Emergency, call for urgent help and start oxygen therapy
7	**SpO2(%) PR(bpm)**<br>**97 107**<br>**Pleth**<br>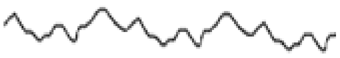	A 9-month baby is having a convulsion (fit). **SCENARIOS**<br>☐1 This is a normal reading, continue routine care<br>☐2 The SpO2 is low<br>☐3 Do nothing, check again in 10 minutes<br>☐4 Poor pleth waveform, check the probe placement and try again<br>☐5 This is an Emergency, call for help and start oxygen therapy
8	**SpO2(%) PR(bpm)**<br>**87 142**<br>**Pleth**<br>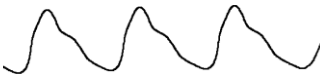	A 2-year-old child comes to hospital with fast breathing.<br>☐1 This is a normal reading, continue routine care<br>☐2 The SpO2 is low, start oxygen therapy<br>☐3 Do nothing, check again in 10 minutes<br>☐4 Poor pleth waveform, check the probe placement and try again<br>☐5 This is an Emergency, call for help and start oxygen therapy
9	**SpO2(%) PR(bpm)**<br>**-- 110**<br>**Pleth**<br>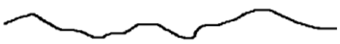	A 12-month child has a cough and fever.<br>☐1 This is a normal reading, continue routine care<br>☐2 The SpO2 is low, start oxygen therapy<br>☐3 Stop oxygen, check again in 10 minutes<br>☐4 Poor pleth waveform, check the probe placement and try again<br>☐5 This is an Emergency, call for help and start oxygen therapy
10	**SpO2(%) PR(bpm)**<br>**99 144**<br>**Pleth**<br>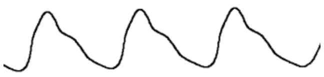	An 1700g newborn baby is on oxygen therapy (1LPM).<br>☐1 This is a normal reading, continue routine care<br>☐2 Reduce oxygen flow rate, the SpO2 is too high for a preterm neonate<br>☐3 Stop oxygen, check again in 10 minutes<br>☐4 Poor pleth waveform, check the probe placement and try again<br>☐5 This is an Emergency, call for help and start oxygen therapy
