# Supplementary material for: Adoption of paediatric and neonatal pulse oximetry by 12 hospitals in Nigeria: a mixed-methods realist evaluation
Source: BMJ Glob Health. 2018 Jun 26;3(3):e000812. doi: 10.1136/bmjgh-2018-000812 (PMC6035503; doi:10.1136/bmjgh-2018-000812)
Supplement: Supplementary data [file bmjgh-2018-000812supp004.pdf]

Interview Guide

Provide Participant Information and obtain Consent.

Welcome: Thank you for taking the time to join us to talk about pulse oximetry use at your hospitals. My name is Hamish Graham, and assisting me is _____________.

Overview: Today we are having a group discussion to learn how the Oxygen project has been going in different hospitals. We want to learn about what it is like to introduce pulse oximetry into a hospital. You are the experts. You have been on the ground for the past year as we have been encouraging nurses to use pulse oximetry. You saw what it was like before we introduced the oximeters. You saw the training. You saw some people embrace oximeters quickly – and others struggle. You have seen things that work well, and things that make it difficult. Today, we want to hear what you have observed. We want to know what you – the experts – think about how introduce pulse oximetry into a ward.

Ground rules: We want you to be completely frank and honest. There are no wrong answers – we want to hear different points of view. We want to hear the good and the bad – what worked well, and what failed. We want to hear stories and examples – but we don't need to know names or personal details.

Recording: We are recording the session because we don't want to miss any of your comments and we won't be able to write fast enough to keep up with you. We will use your names during this discussion. But we will not use your names, or other people's names, when we write our report. We ask that you respect each other's privacy, and do not discuss personal details shared in this discussion.

Any questions?

First question: Let's begin. We have placed name cards in front of each of you, to help me remember each other's names. Let's start by introductions – please share your name and the hospital you have been working with.

Start tape recorder

QUESTIONS

**Q1 – Before intervention**<br>Think back to what things were like before the Oxygen Implementation team brought the pulse oximeters, and before they did oximetry training.<br><br>**What experience did nurses and doctors in your hospital have with pulse oximetry BEFORE the oxygen project came?**	Positive experience<br>Negative experiences<br>Previous Training?<br>Previous Protocols?<br>Who made decisions about oxygen?<br>Examples… Tell me more…
**Q2 – Response to the introduction of pulse oximetry**<br>Think back to when pulse oximetry was first introduced by the Oxygen Implementation Project.<br><br>**What were people's first responses to the pulse oximeters and the training?**	Positive responses<br>Negative responses<br>Why respond differently<br>Were you surprised by responses<br>What did they say about…<br>…Equipment …Training
**Q3 – Current practices**<br>Think back over the past few weeks or month.<br><br>**What do people think and say about pulse oximetry now?**	Examples… Tell me more…<br>How do they feel? Good / bad<br>What do they say? Good / bad<br>Are they confident? Anyone not?<br>What has changed? How?
**Q4 – Barriers & Enablers**<br>One year ago, almost no children had pulse oximetry done. Now most children do – but it has been a gradual improvement. [show data] Every hospital has things that encourage or discourage nurses to regularly use pulse oximetry.<br><br>**What encourages nurses use pulse oximetry routinely in your hospital? Or discourages, makes it inconvenient?**	Examples… Tell me more…<br>What encourages / convenient?<br>What discourages / inconvenient?<br>If you were in charge…<br>Advice for other hospitals…
**Q5 – Concluding**<br>Today we wanted to hear from you about how pulse oximetry is used. You have given us a lot of very valuable information, and lots for us to think about.<br><br>**Of all the things we have discussed today – in your opinion, what is the most important?**	Most important<br>Missed anything
**Q6 – Reflective**<br>At the start we said we wanted to have this discussion group so that we could learn from the experts – from you.<br><br>**What has it been like being part of this Discussion Group?**	Was it useful?<br>Was anyone nervous / scared?<br>Would you do it again<br>Suggestions to make better

Focus Group / Interview Sign In Sheet

Date ________________

Name & Job position	Age (years)	Sex (M/F)	Hospital(s)	Time period at hospital

Participant Information

This study aims to understand how pulse oximetry is adopted into routine clinical practice by staff in paediatric and neonatal wards.

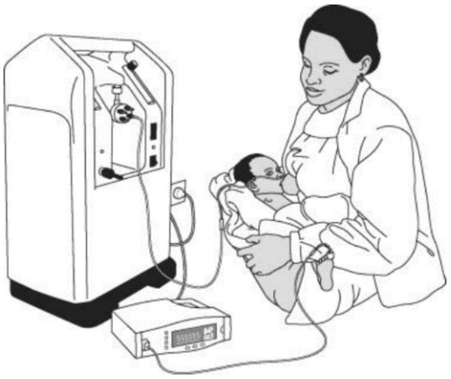

Why is it important?

Hypoxaemia (low blood oxygen levels) is a common and potentially fatal complication of pneumonia and many other severe illnesses of childhood and newborns.

Routine use of pulse oximetry can identify children with hypoxaemia better than relying on clinical signs alone, making it possible to know which children need oxygen therapy – and guiding the clinical use of oxygen. However, many hospitals have faced challenges in adopting pulse oximetry as part of routine clinical practice and we have a limited understanding of these challenges – or the possible solutions.

We want to learn how to effectively introduce pulse oximetry into routine clinical practice in the paediatric and neonatal ward settings. We hope that this information will help staff in other hospitals that are seeking to improve the use of pulse oximetry for children and neonates.

What will the study involve?

This project is being done as part of the Nigeria Oxygen Implementation Project, which seeks to improve oxygen access and use in 12 Secondary Health Facilities in south-west Nigeria (supported by the Bill and Melinda Gates Foundation). Hospitals have all been provided with Lifebox® pulse oximeters and training on how to use it.

This study will gather information from research nurses and clinicians through focus groups and interviews. We will be asking questions about what you have observed about pulse oximetry practice in your hospital. We will be asking about what things have made it challenging to do pulse oximetry routinely, and what things have helped make pulse oximetry a routine part of practice in your hospital.

We will also collect some basic personal information about you (e.g. age, position, sex) to help us with the research project - all information that can identify you will remain confidential. Your information will be de-identified and stored in password-protected computers for a minimum of 3 years or as required by law. Your information will only be used for the purpose of this research project and it will only be disclosed with your permission, except as required by law.

Project Information

TITLE: **Pulse oximetry adoption project**: *Understanding the adoption of pulse oximetry by nurses in Nigerian district hospitals: a mixed-methods study* (ID: 1647681.1).

INVESTIGATORS:

Dr Hamish Graham, Centre for International Child Health, University of Melbourne, Melbourne, Australia (Ph: +61 393454977, E: hamish.graham@rch.org.au)

Prof AG Falade/Dr I Ayede, Department of Paediatrics, University College Hospital Ibadan, Oyo state, Nigeria.

LOCATION:

University College Hospital Ibadan, Oyo state, Nigeria

THE UNIVERSITY OF MELBOURNE

How will the information I provide be used?

We will be presenting the results of this research at forums and publication, and information will be provided in such a way that you cannot be identified, except with your express permission. All data will be de-identified and described in terms of general demographics (e.g. quote may be attributed to “H1P1” = participant 1 at hospital 1).

We will store the data securely and confidentially according to law. There will be no financial reimbursement for participation, and participation is fully voluntary. Your participation, or decision not to participate, will not impact negatively on your role with the Oxygen Implementation Project or the hospital where you are based. You are able to request access to the information that is collected and to request that any information with which you disagree be corrected.

What will I need to do?

We would like to learn from you about how pulse oximetry has been introduced to the hospital where you are based - what has worked well, what are the challenges. Participation in this project requires:

- Participation in a focus group / interview, exploring questions relating to the introduction of pulse oximetry in the hospital where you are based. These will be conducted at a mutually convenient time and place and will last for approximately 1 hour.
  [NB: focus groups are intended for groups of 2-4 research nurses]

- *Optional* – Opportunity to give feedback on the researcher’s findings prior to completion of the study report.

If participation has caused you any distress or worry, please talk to Dr Hamish Graham, Dr A.A. Bakare, Prof A.G. Falade, or Matron. If wish to speak to someone outside the research team, please contact Prof Adesoji Fasanmade - +234 (0)803 328 4877; adesojif@yahoo.com.

If you have any concerns about the way this research is conducted, please contact Dr Hamish Graham.

Name	Dr Hamish Graham
Position	Paediatrician, primary researcher
Telephone	+61 3 93456262
Email	hamish.graham@rch.org.au

THE UNIVERSITY OF MELBOURNE

This research project has been approved by the Human Research Ethics Committee of The University of Melbourne. If you have any concerns or complaints about the conduct of this research project, which you do not wish to discuss with the research team, you should contact the Manager, Human Research Ethics, Office for Research Ethics and Integrity, University of Melbourne, VIC 3010. Tel: +61 3 8344 2073 or Email: HumanEthics-complaints@unimelb.edu.au. All complaints will be treated confidentially. In any correspondence please provide the name of the research team or the name or ethics ID number of the research project.

Consent

I have received information regarding the project, including information on the focus groups and interviews, and have had an opportunity to ask questions.

I understand this study will involve **my voluntary participation in focus groups / interviews to share my perspective on pulse oximetry implementation in my hospital**. Data from these interviews will be collated into reports to inform others who wish to improve the use of pulse oximetry in their hospitals.

I voluntarily agree to participate in a focus group / interview for the project. I understand that I can withdraw my consent to participate at any time.

Name (please print) ____

Signature _______________________________________ Date ________________________

Declaration by Researcher†

I have given a verbal explanation of the research project, its procedures and risks and I believe that the participant has understood that explanation.

Name of Researcher† (please print) ____

Signature _______________________________________ Date ________________________

† An appropriately qualified member of the research team must provide the explanation of, and information concerning, the research project.

The project has Ethics approval from the University of Ibadan/University College Hospital Ethics Committee and Human Research Ethics Committee of The University of Melbourne. If participants have any concerns about the conduct of this research project, contact the Manager, Human Research Ethics, University of Melbourne, ph: 8344 2073; HumanEthics-complaints@unimelb.edu.au. All complaints a treated confidentially.
